# Modulatory Properties of Food and Nutraceutical Components Targeting NLRP3 Inflammasome Activation

**DOI:** 10.3390/nu14030490

**Published:** 2022-01-23

**Authors:** Mattia Spano, Giacomo Di Matteo, Cinzia Ingallina, Donatella Ambroselli, Simone Carradori, Marialucia Gallorini, Anna Maria Giusti, Andrea Salvo, Michela Grosso, Luisa Mannina

**Affiliations:** 1 Department of Chemistry and Technology of Drugs, Sapienza University of Rome, Piazzale Aldo Moro 5, 00185 Rome, Italy; mattia.spano@uniroma1.it (M.S.); giacomo.dimatteo@uniroma1.it (G.D.M.); cinzia.ingallina@uniroma1.it (C.I.); donatella.ambroselli@uniroma1.it (D.A.); andrea.salvo@uniroma1.it (A.S.); luisa.mannina@uniroma1.it (L.M.); 2 Department of Pharmacy, “G. d’Annunzio” University of Chieti-Pescara, Via dei Vestini 31, 66100 Chieti, Italy; marialucia.gallorini@unich.it; 3 Department of Experimental Medicine, Sapienza University of Rome, Piazzale Aldo Moro 5, 00185 Rome, Italy; annamaria.giusti@uniroma1.it; 4 Department of Molecular Medicine and Medical Biotechnology, School of Medicine, University of Naples Federico II, CEINGE-Biotecnologie Avanzate, 80131 Naples, Italy; michela.grosso@unina.it

**Keywords:** NLRP3 inflammasome, modulation activity, food, nutrients

## Abstract

Inflammasomes are key intracellular multimeric proteins able to initiate the cellular inflammatory signaling pathway. NLRP3 inflammasome represents one of the main protein complexes involved in the development of inflammatory events, and its activity has been largely demonstrated to be connected with inflammatory or autoinflammatory disorders, including diabetes, gouty arthritis, liver fibrosis, Alzheimer’s disease, respiratory syndromes, atherosclerosis, and cancer initiation. In recent years, it has been demonstrated how dietary intake and nutritional status represent important environmental elements that can modulate metabolic inflammation, since food matrices are an important source of several bioactive compounds. In this review, an updated status of knowledge regarding food bioactive compounds as NLRP3 inflammasome modulators is discussed. Several chemical classes, namely polyphenols, organosulfurs, terpenes, fatty acids, proteins, amino acids, saponins, sterols, polysaccharides, carotenoids, vitamins, and probiotics, have been shown to possess NLRP3 inflammasome-modulating activity through in vitro and in vivo assays, mainly demonstrating an anti-NLRP3 inflammasome activity. Plant foods are particularly rich in important bioactive compounds, each of them can have different effects on the pathway of inflammatory response, confirming the importance of the nutritional pattern (food model) as a whole rather than any single nutrient or functional compound.

## 1. Introduction

### 1.1. The NLRP3 Inflammasome

**Figure 1:**
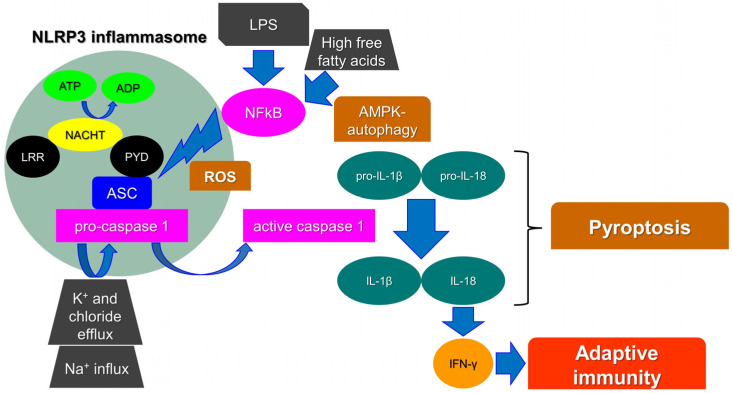
NLRP3 activation and the main players involved in pyroptosis. Leucine-rich repeat (LRR), pyrin domain (PYD), interferon-gamma (IFN-γ), interleukin (IL), reactive oxygen species (ROS), lipopolysaccharide (LPS), NOD-, LRR- and pyrin domain-containing protein 3 (NLRP3), caspase-recruitment domain (ASC), nuclear factor kappa-light-chain-enhancer of activated B cells (NF-kB), AMP-activated protein kinase (AMPK), adenosine diphosphate (ADP), adenosine triphosphate (ATP).

Inflammasomes are key intracellular multimeric protein complexes, able to initiate inflammatory signaling through sensor receptors, defined as pattern-recognition receptors (PPR). PPRs recognize pathogen-associated molecular patterns (PAMPs), or damage-associated molecular patterns (DAMPs) generated by endogenous stress stimuli. The signal transduction continues intracellularly through an adaptor protein and an effector enzyme that cause the maturation and the secretion of pro-inflammatory cytokines. As a result, the inflammasome activation ends up with the production of caspase-1, a major mediator in the inflammatory adaptive immune cell response [1]. Pro-interleukin 1β (pro-IL-1β) and pro-IL-18 are cleaved by active caspase-1 into their biologically active form to trigger endothelial cell responses, such as vasodilatation, which allows the extravasation of immune cells, or acts to control hypotension, fever, and pain threshold. In particular, IL-18 is crucial for interferon-gamma (IFN-γ) production and thus for adaptive immunity [2]. Finally, caspase-1 activation stimulates a unique pro-inflammatory cell death process, namely pyroptosis. Pyroptosis triggers intracellular pathogens to escape from their replicative environment, leading to their exposition to immune factors and thus allowing the immune system to face infections (Figure 1) [3].

Among other PPRs, leucine-rich repeat (LRR)-containing proteins (NLR) family members (NLRP1 and 3) are widely known as components of inflammasomes. In particular, it has been reported that the NLRP3 inflammasome is crucial for adaptive cell responses towards viral, bacterial, and fungal infections. Nevertheless, when dysregulated, it has been associated with a variety of inflammatory or autoinflammatory disorders, such as diabetes, gouty arthritis, liver fibrosis, Alzheimer’s disease (AD), respiratory syndromes, atherosclerosis, and cancer initiation [4]. The NLRP3 protein consists of a C-terminal leucine-rich repeat (LRR) domain and an amino-terminal pyrin domain (PYD). The central domain is a nucleotide-binding, oligomerization domain (NACHT domain). The interaction between the PYD domain with the caspase-recruitment domain (ASC) facilitates the initiation of the inflammasome assembly. In parallel, the NACHT domain functions as an ATPase and the amount of ADP produced allows NLRP3 oligomerization and activation [5]. Finally, the LRR domain functions as a signal effector; although, its role is still not fully understood. It has been highlighted that the NLRP3, lacking the LRR domain, can be fully activated via the canonical inflammasome pathway. It has been therefore hypothesized that it may act as an auto-inhibitor, thus controlling aberrant signal amplification and participating in a protective mechanism, avoiding autoinflammation [6].

Many molecular mechanisms/compounds have been widely proposed for the activation of NLRP3, involving, among others, biological, physical, chemical, and metabolic agents, such as uric acid crystals, cholesterol, free fatty acids and lipids, β-amyloid (BA) protein, aluminum hydroxide, high intracellular glucose, and high levels of reactive oxygen species (ROS). Others factors, such as heme, ionic flux, lipopolysaccharide (LPS) toxin, pathogen-associated RNA, and ceramides, are also included [7]. Likewise, Ca^2+^-related signaling, Na^+^ influx, and K^+^ and chloride efflux have been reported as crucial events in the NLRP3 inflammasome [8]. As a matter of fact, a recently identified component of the NLRP3 inflammasome, namely NEK7 (NIMA-related kinase 7), requires K^+^ efflux for inflammasome assembly. It has been proposed that the activation of NLRP3 requires two steps. Firstly, the mitotic kinase NEK7 binds to NLRP3; although, this complex could not be sufficient for NLRP3 activation. Indeed, the inflammasome oligomerization requires ATP binding and thus the conversion of NACHT from an inactive to an active conformation [9]. Reactive oxygen species (ROS) production, mainly from mitochondria, has also been reported as an initiator of the activation of the NLRP3 inflammasome. Many studies have shown that NLRP3 agonists generate ROS in different cell types. As an example, fatty acid caused by a high-fat diet activates the NLRP3 inflammasome through the molecular axis AMPK–autophagy–NF-kB-ROS [7]. Moreover, Amyloid β, post-translational modifications of NLRP3, and non-canonical inflammasome activations have been reported as triggers for the NLRP3 inflammasome [10,11,12].

### 1.2. Food and Inflammation (Diet and Inflammation)

The emerging role of chronic inflammation as a determinant in the progress of the major degenerative diseases typical of modern society has promoted research to comprehend the influence of nutrition and dietary patterns on inflammatory markers. Dietary intake and nutritional status represent important environmental factors, which can modulate metabolic inflammation. In recent years, research has advanced significantly to achieve understanding of the impact of dietary components on metabolic inflammation, in the context of chronic infirmities, such as obesity, type-2 diabetes (T2D), cardiovascular diseases (CVD), and cancer [13]. Inflammatory and innate immune responses, provoked by pathogen-associated and other danger-associated signals, appearing during infections, result in the activation of cytosolic inflammasomes. Inflammasome signaling mainly furnishes a host innate immune defense against a broad range of microbial infections, including influenza virus [14,15]. Furthermore, NLRP3 inflammasome can also be activated by different endogenous risk agents, such as palmitic acid, amyloid β, and cholesterol crystals [16]. So, NLRP3 inflammasome intervening in such responses is associated with the development of several lifestyle-related chronic diseases, characterized by persistent inflammation [17].

In the mid-1900s, an important concept was emerging about the relationship between the immune and metabolic response systems, indicating that insulin resistance (IR), glucose intolerance, dyslipidemia, and other metabolic abnormalities occur in the course of an infection. Later, in the 1980s, a reduced binding capacity of insulin to its receptor in isolated blood cells was found in human patients affected by acute infection [18]. The network that connects metabolic and immune functions has been structured in relation to a lifestyle that was quite different from that of today, so that the current metabolic overload induces a low-grade chronic inflammatory state, for which some authors have recently proposed the term “metaflammation”. This term is used to describe the chronic low-grade inflammation orchestrated by metabolic cells in response to excess nutrients and energy. Metaflammation plays a pivotal role in the development and systemic expansion of the metabolic disease. White adipose tissue is likely to be the primary site of the metaflammation development; although, progressively, other metabolic tissues, such as liver, pancreas, and gut cells, get involved, affecting the metabolic homeostasis [13,19,20].

The strong link between nutrient sensing and immune signaling is justified by an evolutionarily conserved crosstalk pathway between immune and metabolic mediators. The functional units that control key metabolic and immune functions in higher organisms have evolved from common ancestral structures. Some invertebrates, such as the fruit fly *Drosophila melanogaster* or the nematode *Caenorhabditis elegans*, gave similar evidence of a conventional cellular crosstalk between immune and metabolic organs. Basically, cytokines act as metabolic hormones in the adaptation to nutrient fluctuations [13]. In *Drosophila*, for example, the body fat acts as the liver, adipose, and immune system, serving both the functions of nutrient storage and body defense [20]. During the course of evolution, a conservative, similar structure in an ancient mammalian ancestor differentiated into distinct metabolic organs and immune ones, as we essentially have in modern mammals, including humans. The principal framework of this translation from an adaptative to a maladaptive state is resumed by three integrated systems—Eiger, the *Drosophila* orthologue of TNF, and its receptor Wengen (in *Drosophila*) or TNFR (human); insulin and its receptors (dILP in *Drosophila*, insulin receptor insR in human); the TLR signaling pathways (Toll receptor in *Drosophila*). TNF and TLR signaling block insulin pathway or production through JNK activation and MyD88, from flies to humans, whereas activation results in abnormal metabolic homeostasis adaptation, which leads to a chronic metabolic inflammation. As a result, immunometabolic diseases often appear as clusters and promote ageing, disability, and premature death [13].

It has been shown that an excess of fat mass, especially the visceral type, associated with a diet rich in saturated fats and simple sugars and low in fiber and micronutrients (minerals and vitamins), which is the current Western diet, is able to promote a chronic low-grade inflammatory state, which, albeit involving molecules and signals common to the classic inflammatory response, recognizes the primary trigger in the metabolic overload [21]. Conversely, obesity, by inducing chronic low-grade activation of inflammatory pathways, is linked to the development of IR and T2D [22]. In relation to the different organ meiopragia, genetically determined, the metaflammation can manifest itself in pathologies, such as tumors, neuro- and cardio-vascular diseases, neurodegenerative diseases (AD), or metabolic diseases (metabolic syndrome, T2D, non-alcoholic fatty liver disease (NAFLD)). These pathologies are characterized by a variable increase in serum biomarkers level of inflammation, especially TNF-α, IL-1β, IL-6, PCR, fibrinogen, intercellular (ICAM1), and vascular (VCAM1) adhesion molecules, etc. These biomarkers were also shown to be significantly related to the risk of T2D, cardiovascular disease and cancers in healthy subjects [23].

Moreover, particular interest has been directed to IL-1β-mediated inflammation because of the strong inter-relationship between dietary fats, metabolic stressors, and NLRP3-mediated inflammation [24]. Metabolic stress, in the form of obesity, saturated fatty acids (SFAs), cholesterol, reactive oxygen species, and/or uric acid promote IL-1β signaling [25]. IL-1β disrupts cellular metabolism by interrupting a range of signaling pathways, including insulin sensitivity, lipid metabolism, and adipogenesis [26]. It is now widely accepted that IL-1β signaling plays a key role in the development of obesity, IR, and T2D risk [27,28,29,30,31].

### 1.3. Role of Diet in Inflammatory Response

During the feeding/fasting cycle, in physiological conditions, an intermittent, non-specific, low-grade inflammatory response occurs at the level of “metabolic tissues” (adipose, muscle, and liver tissue), which results in a transient increase in some inflammatory proteins/cytokines in the serum. This increment reaches its maximum peak during the absorption phase (post-prandial) and then gradually decreases in about 2 h, when the nutrients have been distributed, metabolized, and/or accumulated in the respective cellular sites [32]. The inflammatory response is amplified in overeating conditions (hyperlipidic diet, excess of saturated fats and simple carbohydrates, and low intake of fiber, vitamins, and antioxidant compounds), in obesity, and in diabetes—when the metabolic overload generates a “traffic jam” of the physiological metabolic pathways, progressive recruitment and activation of immune-competent cells, such as macrophages, mast cells, and T lymphocytes, occurs [33]. This results in the establishment of a vicious circle in which the physiological quiescent phase of the inflammatory response is incomplete and generates a pro-inflammatory milieu that impairs the metabolic functions [34]. On the contrary, a food pattern adherent to the Mediterranean dietary model is associated with a reduction in serum concentration of inflammatory biomarkers [35]. The complex mechanisms through which this inflammatory response is induced by the diet are gradually emerging. It was found that both stages of IL-1β priming and NLRP3-mediated IL-1β activation may be affected by the nutritional environment. One of these mechanisms appears to be associated with the quality of the diet, that is, the excess or lack of specific nutrients intake [28,36].

### 1.4. Fatty Acids and Inflammatory Response

Experimental data showed that the quantity and quality of fats influences the acute inflammatory response to a single meal. The study revealed that SFAs content and the ratio among polyunsaturated fatty acids (PUFAs) from ω-3 to ω-6 have proved to be the major determinants of the extent of postprandial inflammatory response [36,37]. In particular, it has been observed that high meal SFA increased inflammatory indices; contrarily, high ω-3/ω-6 PUFA ratio decreased them [36,37].

Fatty acids can affect the inflammatory response either through the synthesis of eicosanoids that regulate the transduction of signals at cell membrane or cytoplasmic level, by modulating the activity of transcription factors involved in the inflammatory process.

An examination of data from the National Health and Nutrition Examination Survey (NHANES 99-00) found that levels of SFAs in serum phospholipids of male civil servant workers (40–69 years) positively correlated with some inflammatory markers, such as HS-CRP (high sensitivity C-reactive protein) and fibrinogen; by contrast, phospholipid PUFA levels were inversely associated with HS-CRP [32,36]. It has been recently shown in a C57BL/6 male mice model that consuming a high-fat diet, enriched with SFAs, induces especially adipose IL-1β inflammation and insulin resistance. However, if SFAs are replaced with monounsaturated fatty acids (MUFAs), an attenuation of NLRP3-mediated inflammation is observed [16,28].

In vitro studies have shown that SFAs, such as lauric, myristic, and palmitic acids, are able to activate the Toll-like receptors (TLR) of adipocytes and macrophages [28]. The SFA-TLR interaction determines the activation of c-jun N-terminal kinase (JNK), the inhibitor of kappa B kinase (IKK), and protein kinase R (PKR), to which it follows that:

- Phosphorylation of insulin receptor substrate-1 (IRS-1) in serine and its degradation and elimination through the ubiquitin pathway, thus blocking insulin signal transmission;
- Activation of the transcription factor NF-kB, by its dissociation from the cytoplasmic inhibitor (IkB) and transfer to the nucleus, with over-regulation of the pro-inflammatory gene expression.

In conclusion, SFAs impair insulin signaling through TLR4, activating the NF-κB pathway and NLRP3 inflammasome that promotes the conversion of pro-IL-1β into mature IL-1β [16,31,32]. In a clinical study, the relationship between SFAs and inflammation biomarkers has been demonstrated in subjects with excess weight, in which the ω6:ω3 ratio is directly related to the concentrations of IL-6, CRP, and adhesion molecules; in addition, the inhibition of IRS-1 and the reduction in adiponectin levels induce IR, thus increasing the risk of the metabolic syndrome [21]. Additionally, trans-fatty acid (TFA) consumption has been found to be positively associated with markers of systemic inflammation. In the Harvard Nurses’ Health Study, it emerged that TFA intake was positively associated with IL-6 and HS-CRP in women with higher BMI (body mass index) and serum HS-CRP level [38]. Partially hydrogenated oils (PHO) are the main source of industrially produced TFA. PHO is an ingredient in different foods, including margarine and vegetable shortening, but also in baked foods (crackers, biscuits, pies), or in those fried in semi-hydrogenated oils/fats. However, there are also many foods that naturally contain TFA, such as dairy products and meat from ruminant animals. A review of observational and interventional data concluded that the TFA pro-inflammatory effects (increased TNF-α, IL-6, and HS-CRP) were associated with markers of vascular endothelial dysfunction and were most evident when compared with the effects of cis-unsaturated fatty acids [38]. Controlled trials and observational studies provide concordant demonstration that the TFA intake from PHO adversely affects multiple cardiovascular risk factors and contributes significantly to increased risk of CHD events [39]. Several experimental and observational studies in humans have demonstrated the potential benefits of replacing SFAs with unsaturated fatty acids, such as oleic acid and PUFAs. These studies have shown an inverse association between consumption of ω-3 PUFA and systemic markers of inflammation, such as TNFs and ILs [40]. ω-6 PUFA consumption shows variable effects on inflammation. Both anti-inflammatory and pro-inflammatory effects have been described [41], suggesting how the interaction of ω-6 PUFAs and their lipid mediator derivatives in the context of inflammation is complex and still not properly understood. For example, in overweight subjects, the serum concentration of ω-6 PUFAs (α-linoleic acid (ALA), AL, and AA) has been shown to be inversely related to IL-6 levels [41]. ω-3 PUFAs (ALA, eicosapentaenoic acid (EPA) and especially docosahexaenoic acid (DHA)) have much more marked effects than ω-6 PUFAs in reducing the expression of numerous pro-inflammatory cytokines, such as IL-6, TNF-α, IL-18, sICAM-1, sVCAM-1, sE-selectin, and HS-CRP. ω-3 PUFAs suppress NLRP3 inflammasome in obese subjects through downregulation of inflammasome gene expression in adipocytes and macrophages [28]. It has been shown that stimulation of macrophages with ω-3 PUFAs (at a dose of 20 μM) was able to suppress NLRP3 inflammasome activation and to inhibit subsequent caspase-1 activation and IL-1β secretion [42,43].

Furthermore, the ω-3 PUFAs, by binding to the G-protein-coupled receptor 120 (GRP120—a membrane receptor sensitive to fatty acids, expressed at the level of macrophages), block the NFκB activation pathway. Finally, EPA would be able to inhibit the activity of the Δ-6 desaturase enzyme, thus reducing AA formation [42,44]. Some studies indicate that dietary consumption of MUFAs, oleic acid in particular, may have anti-inflammatory effects. Studies suggest that dietary MUFAs can reduce IL-1β-mediated adipose dysfunction and insulin resistance via preservation of AMP-activated protein kinase (AMPK) activity which, in turn, quenches NLRP3 inflammasome activation [30,45,46].

### 1.5. Carbohydrates and Inflammatory Response

Diets with relatively high glycemic index (GI) and glycemic load (GL) have been linked to elevated risk of T2D, coronary heart disease, and stroke, particularly among overweight individuals [47]. GI and GL are associated with an increase in the plasma concentration of inflammatory biomarkers, such as IL-6, HS-CRP, and IL-18 [36]. There are convincing data suggesting that NLRP3 inflammasome is fundamental in the deleterious effect observed in chronic hyperglycemia and oxidative stress. Hyperglycemia is associated with both conditions of oxidative stress and inflammatory state and promotes mitochondrial metabolism in β cells which enhances the production of ROS, thus inducing NLRP3 inflammasome activation and then IL-1β production [48].

Recent clinical trials indicate that targeting the prototypic pro-inflammatory cytokine IL-1β ameliorates the outcomes of cardiovascular disease, which is the first cause of death in T2D patients. Several T2D-related metabolic factors, including reactive oxygen species, glyco/lipoxidation end products, and cholesterol crystals, have been involved in the pathogenesis of diabetes complications (diabetic kidney disease and diabetic retinopathy) and in the progression of atherosclerosis and NAFLD [49].

High levels of glucose and non-esterified fatty acids (NEFAs) can cause oxidative stress due to both the decoupling of oxidative phosphorylation and to the increased mitochondrial β-oxidation. Lee et al. found that hyperglycemia-induced elevated mitochondrial reactive oxygen species in myeloid cells of T2D patients are associated with increased production of inflammasome-dependent cytokines IL-1β and IL-18 [50]. Some data also showed that inhibition of AMP-activated protein kinase can exacerbate ROS-dependent NLRP3 activation [50]. It was recently pointed out that NLRP3 might directly sense the presence of increased ROS production (mitochondrial superoxide anion radical (O_2_^•−^), hydrogen peroxide (H_2_O_2_)) by normal or malfunctioning mitochondria, or indirectly by other activators of NLRP3. In particular, it was suggested that increased ROS are sensed by a complex of redox-dependent inhibition of thioredoxin (TRX) and TRX-interacting protein (TXNIP) and cause the dissociation of the complex described above. Following an increase in cellular ROS concentration, this complex dissociates and TXNIP binds to the LRR region of NLRP3, with the consequent NLRP3 activation [51].

Chronic hyperglycemia (associated or not with increased triglycerides and NEFAs) promotes the formation of advanced glycation end-products (AGEs). The interaction between AGEs and receptors for advanced glycation end-products (RAGEs), the activation of the polyols pathway and the auto-oxidation of glucose lead to increased production of ROS which, in turn, induce the activation of transcription factors, such as NF-kB [52]. The result is an increase in inflammatory biomarkers, such as HS-CRP, IL-6, IL-1β, IL-8, IL-18, and TNF-α, matrix metalloproteinases, and markers of endothelial dysfunction (VCAM-1, ICAM-1 and E-selectin). Redox signaling molecules, such as ROS, mediate NLRP3 inflammasome activation, while ROS inhibitors suppress NLRP3 inflammasome-mediated inflammation [52], indicating the vital role of oxidative stress on inflammasome activation that can be prevented by supplementation with antioxidants, such as vitamin C, E, lipoic acid, phenolic compounds, and other bioactive compounds found in plant foods [48,53].

Consumption of whole grains, through increased fiber intake, is associated with a reduction in serum concentrations of inflammation biomarkers (HS-CRP, IL-6, Il-1 β); although, the mechanisms still need to be elucidated, and a synergistic effect of fiber, minerals, vitamins, or phytochemicals, such as lignans and phenolic acids, could take place [21,36].

Several studies in recent years have demonstrated the role of specific nutrients in the primary or secondary prevention of inflammation-related diseases, such as metabolic syndrome and diabetes. In this regard, some nutraceuticals and bioactive compounds (polyphenols, flavonoids, carotenoids, curcumin, resveratrol, etc.) have been widely studied and have been recognized for their ability to inhibit the synthesis of TNF-α in monocytes and macrophages and downregulate the expression of TLR2 and TLR4 in human monocytes [1].

However, due to the reciprocal synergistic or antagonistic interactions between nutrients and/or other food compounds (antinutrients and/or phytochemicals), it is clear that, in order to understand the role of diet in the modulation of the inflammatory state and oxidative stress, it is necessary to evaluate the nutritional pattern in its entirety. It was found that diet characterized by an abundant consumption of vegetables, fruit, nuts, olive oil, legumes, and fish, with moderate amounts of alcohol and low intake of red meat, processed meats, refined grains, and dairy products, has a protective effect against immunometabolic diseases [49]. These aspects correspond to the characteristics of the Mediterranean dietary pattern. Indeed, several studies have shown an inverse association between the degree of adherence to the diet Mediterranean and serum levels of IL-6, CRP, and TNF-α [35].

## 2. Materials and Methods

**Figure 2:**
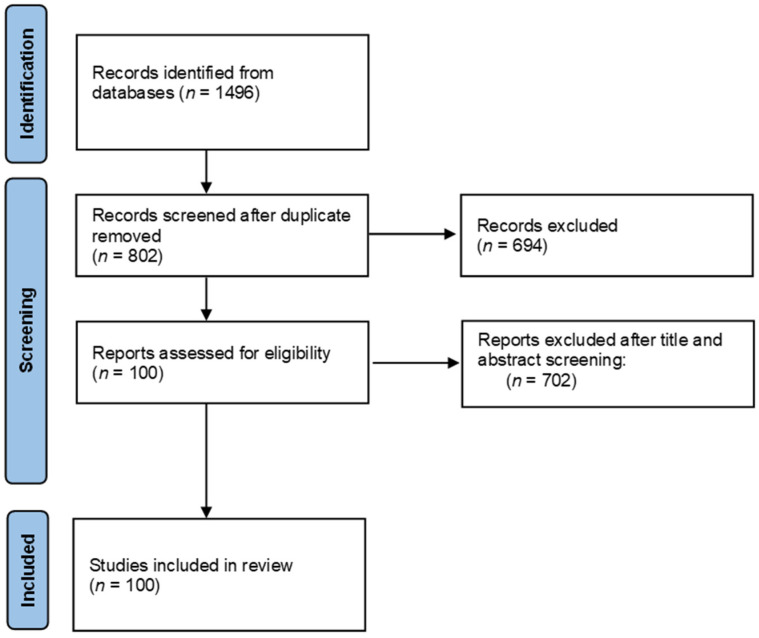
Preferred Reporting Items for Systematic Reviews and Meta-Analyses (PRIMSA) flow chart.

The literature search was carried out in August 2021 in official scientific databases, using combinations of keywords “NLRP3”, “NLRP3 inflammasome”, “food”, “nutrients”, and “bioactive compound” in the literature published during the last five years. Study selection and data were collected according to the 2009 Preferred Reporting Items for Systematic Reviews and Meta-Analyses (PRISMA) guidelines [54], Figure 2.

## 3. Food Components as NLPR3 Modulators

### 3.1. Polyphenols

Polyphenols are secondary metabolites produced by the metabolic processes of plants. Although more than 8000 polyphenols have been identified up to now, all these compounds arise from a common intermediate—phenylalanine or shikimic acid. Generally, they occur in conjugated forms, with one or more sugar residues, organic acids, amines, lipids, and other phenols [55]. Polyphenols may be classified into different groups based on their phenol rings number or on the structural elements that bind these rings to one another. The main classes include phenolic acids, flavonoids, stilbenes, and lignans. In recent years, a great interest on the potential health effects of polyphenols has been developed, since these molecules have shown to be mainly characterized by antioxidant and anti-inflammatory activities, proving to be very effective in the prevention and reduction in tumors, chronic inflammations, diabetes, aging, and infections [56]. The anti-inflammatory activity of polyphenols has also been demonstrated with the inhibition of the NLRP3 inflammasome activation. In the present paragraph, the anti-NLRP3 inflammasome activity of several polyphenols is discussed, by dividing the discussed compounds basing on their polyphenol class. A summary list of all the discussed food derived molecules is presented in Table 1.

#### 3.1.1. Phenolic Acids

Phenolic acids are one of the main compounds of polyphenol class. Phenolic acids are characterized by a phenolic ring with a carboxylic acid function and can be further divided into two main groups, benzoic acid or cinnamic acid derivatives, based on the C1–C6 or C3–C6 backbones [132]. Several compounds belonging to the phenolic acids family have shown to possess interesting antioxidative and anti-inflammatory properties [56,133]. The modulating activity against the NLRP3 inflammasome complex has also been widely studied [1].

Sinapic acid is a hydroxycinnamic acid widely found in many foodstuffs, such as vegetables, spices, citrus, berry fruits, cereals, oilseed crops, wine, and vinegar. Sinapic acid has shown antioxidant and anti-inflammatory properties [134], as well as a modulating activity against the NLRP3 inflammasome complex in mouse models with chemically induced colitis [57]. The monitoring of NLRP3 inflammasome protein levels in the inflamed colon tissue of Kunming mice (colitis model), by Western blot analysis, after sinapic acid treatment for 7 days, have shown a reduction in the amounts of NLRP3, ASC, IL-1β, and caspase-1 proteins. In particular, the administration of 50 mg/kg of sinapic acid had a higher effect than 10 mg/kg.

Ferulic acid is an another hydroxycinnamic acid commonly found in fruits, vegetables, grains, beans, leaves, seeds, nuts, grasses, flowers, and in some plants, such as corn and wheat, and in the spice turmeric [135]. A total of 24 adult male Wistar rats with kidney injury induced by methotrexate were treated with 25 or 50 mg/kg of ferulic acid for 15 days [58]. Both doses of ferulic acid have produced an amelioration of NLRP3 and caspase-1 proteins expression, and subsequently a reduction in the IL-1β levels in the rat kidneys.

Chlorogenic acid is another phenolic acid, widely studied for its anti-inflammatory properties [136]. Chlorogenic acid can be found in foods, such as apples, coffee beans, eggplants, grapes, kiwi fruits, pears, plums, potatoes, tea, and tomatoes. This molecule has demonstrated an inhibitory effect against the NLRP3 signaling pathway, decreasing the protein levels of NLRP3, ASC, caspase-1 p45, and caspase-1 p20 in the colon tissue of BALB/c mice with induced colitis [59]. The NLRP3 expression reduction in RAW264.7 cells stimulated by LPS and adenosine triphosphate, resulting in the decreased secretion of IL-1β and IL-18, was also observed.

Lastly, among cinnamaldehyde-related compounds, only cinnamaldehyde and 2-methoxycinnamaldehyde limited the expression of NLRP3 and pro-IL-1β at 25–100 µM, whereas cinnamic acid, cinnamyl alcohol, cinnamyl acetate, and α-methylcinnamaldehyde were ineffective, thus demonstrating the importance of the propenal group in the side chain [137,138].

#### 3.1.2. Flavones and Flavanones

Among flavones, apigenin, largely present in common fruits and vegetables, such as parsley, onions, oranges, tea, etc., has shown to be effective in reducing NLRP3 inflammasome activation in two different studies. In the first one, in vivo and in vitro models of high-fat diet-induced non-alcoholic fatty liver disease (HFD-induced NAFLD) have been treated with apigenin [60]. In particular, mice were administered by gavage with 50 mg/kg apigenin (4 mg/kg HED); whereas, for in vitro studies, Hepa1–6 cells were exposed for 24 h with 16 and 32 µM apigenin. In both assays, an important liver reduction in NLRP3, ASC, pro-caspase-1, and caspase-1 (measured by PCR), together with a reduction in ROS production was obtained by inhibiting xanthine oxidase. In the second study [61], 30 and 50 µM apigenin showed to be effective in reducing NLRP3 protein expression in ISO-HAS human endothelial cells treated with the pro-inflammatory agent TMAO (trimethylamine *N*-oxide). Isoorientin, a flavone identified in Gentiana roots, has shown to possess anti-NLRP3 inflammasome activation in in vivo and in vitro models of hyperuricemia by using 5 and 10 mg/kg (0.4 and 0.8 mg/kg HED) dosages in male ICR mice for in vivo studies and 25, 50, 100, 200, and 400 µM isoorientin for in vitro assays (MXC207 cells). In both cases, a dose-dependent reduction in inflammasome expression was observed, through the inhibition of xanthine oxidase activity and interleukin release [62]. In a very similar way, the anti-inflammasome activity in hyperuricemia male Sprague Dawley mice model has also been demonstrated for chrysin flavone, used at 50, 100, and 150 mg/kg oral doses (4, 8, and 12 mg/kg HED) for 4 weeks [63]. Luteolin preventive effects in spinal cord ischemia–reperfusion injury (SCII) have been demonstrated in an in vivo study where the treatment of male Sprague Dawley mice with intragastric injections of 50 and 100 mg/kg doses (4 and 8 mg/kg HED) of the considered flavone for 14 days has shown to reduce, with respect to non-treated mice, the incoming of induced SCII. In particular, the reduction in this event has been associated with the reduction in NLRP3, IL-1β, and IL-18 expression by ELISA assay [64].

Regarding flavanones, hesperidin methylchalcone and naringin have been demonstrated to be effective in reducing NLRP3 inflammasome activation. In particular, hesperidin methylchalcone, particularly present in citrus fruits, has shown to reduce the NLRP3, ASC, pro-caspase-1, and pro-IL-1β mRNA expression in induced gout arthritis Swiss mice models when orally administrated at 30 mg/kg (2.4 mg/kg HED) [65]. In a model of DSS-induced ulcerative colitis in male C57BL/6 mice [66], naringin has shown to reduce NLRP3, ASC, caspase-1, and IL-1β expression in the colon tissue in a dose-dependent way, when administrated at 25, 50, and 100 mg/kg (2, 4, and 8 mg/kg HED) for 7 days.

#### 3.1.3. Flavonols

Among polyphenols, quercetin, largely present in several vegetables and fruits, has been the most considered compound for the study of its anti-NLRP3 inflammasome activity. In particular, in an alcohol-induced acute liver injury in male SPF-Wistar mice, the oral administration of 100 mg/kg quercetin (8 mg/kg HED) for 14 days caused a reduction in the injury evolution, thanks to a lower expression of inflammasome factors including ROS, NF-κB, NLRP3 inflammasome, IL-1β, and IL-18, measured by ELISA assays in the liver tissue [67]. The same quercetin dosage described in the previous study has been applied for 16 days in high fat treated ApoE^−/−^ mice to observe the potential reduction in atherosclerotic inflammation, where NLRP3 inflammasome is largely involved. In the study, an important decrease in pro-IL-1β and IL-1β was registered [68]. The effect of quercetin in reducing inflammasome activation has also been demonstrated for neurodegenerative models [69], since aging mice treated with 35 and 70 mg/kg (2.8 and 5.6 mg/kg HED) of quercetin for 4 weeks have shown a dose-dependent increase in the cognitive functions, together with an important reduction in NLRP3 activation factors expression by Western blot analysis. An in vitro assay on *E. coli*-infected cells has also been carried out to demonstrate the anti-inflammasome activity of quercetin during infection. In particular, the 12 h treatment with 200 µM quercetin before infection has shown to strongly reduce NLRP3, caspase-1, and IL-1β expression, together with an enhanced ROS scavenger activity [70]. Similarly, a quercetin saturated derivative, dihydroquercetin, has shown to be active against NLRP3 inflammasome in alcoholic liver steatosis in vivo (male C57BL/6 mice) and in vitro (human hepatoma cells HepG2) models [71]. Other two flavonols largely present in tea, vegetables, oranges and wine, namely kaempferol and myricetin, have been shown to be effective in reducing NLRP3 inflammasome. In particular, kaempferol has been used in an in vivo induced hepatotoxicity male C57BL/6 mice model, causing a dose-dependent decrease in NLRP3 inflammasome activation factors (IL-1β, TNF-α, IL-6) in the liver and in the blood when administrated at 30 and 60 mg/kg (2.4 and 4.8 mg/kg HED) dosages for 7 days [72]. It is noteworthy that, in this work, kaempferol has been extracted from a Chinese medical plant but, since its purity has been measured >98%, the activity can be asserted to the single molecule as it is. The same inflammasome inhibition has been observed for myricetin, when its activity has been considered in a high-fat diet model for the simulation of human T2D symptoms in male Wistar mice treated with oral doses of 20 mg/kg (1.6 mg/kg HED) myricetin for 4 weeks [73].

#### 3.1.4. Other Phenolics

Curcumin is the main active compound of turmeric and its anti-inflammatory activities have been largely demonstrated in many inflammatory diseases, in which the inhibition of NLRP3 inflammasome represents an important mechanism of action [139]. This activity has also been observed in a chronic unpredictable mild stress (CUMS) mice model for the simulation of depression pathology, that also involves inflammatory process. In particular, during inducing CUMS in mice, the treatment with 100 mg/kg (8 mg/kg HED) curcumin daily dosages for 4 weeks has caused a reduction in CUMS symptoms in curcumin-treated mice, with respect to untreated ones. This symptom reduction has been accompanied by a strong decrease in NLRP3 inflammasome factors in the hippocampus, namely IL-1β, IL-6, and TNF-α, measured with Western blotting analysis [74]. Interestingly, curcumin supplementation has also been studied in a randomized double-blind clinical study to investigate its anti-inflammatory effects in hemodialysis patients [75]. In this study, half of the considered hemodialysis patients have received, 3 times a week for 12 weeks, a beverage containing 2.5 g of turmeric (95% curcumin) after dialysis. At the end of the trial, patients who received curcumin supplementation have shown lower expression levels of blood NLRP3 inflammasome markers (NF-kB, NLRP3, and IL-1β) with respect to non-curcumin-treated ones, underlining the potential anti-inflammasome activity. 6-Shogaol is one of the main pungent chemical constituents of ginger roots and its anti-NLRP3 activation has been demonstrated in an in vitro assay where cell calcification has been induced in human artery smooth muscle cells with high dosage of glucose. The 14-day treatment of cell cultures with 6-shogaol drastically reduced the expression of NLRP3, caspase-1, and IL-1β [76]. The potent anti-inflammatory activity of 6-shogaol has also been demonstrated when the NLRP3 inflammasome has been activated in human THP-1 monocytes [77]. In this study, 5, 10, 20, and 40 µM of 6-, 8-, and 10-shoagol/gingerol (all present in ginger roots) have been used to treat cell cultures, resulting in a higher dose-dependent decrease in NLRP3 and IL-1β levels when 6-shoagol was used. Pterostilbene, a stilbenoid compound largely present in grapes and blueberries, has shown to be largely effective in inhibiting inflammasome in acute liver failure, allergic contact dermatitis, and hyperuricemia models. In particular, in an acute liver failure model obtained by treating female BALB/c mice with lipopolysaccharide and D-galactosamine, the concurrent intraperitoneal administration of 50 mg/kg/12 h (4 mg/kg HED) pterostilbene for 1 day determined an important decrease in IL-1β, IL-6, caspase-1, TNF-α, and NLRP3 protein [78]. Similar effects, together with ROS reduction, have been observed when 500 mg/kg (40 mg/kg HED) dosage of pterostilbene were injected for 2 weeks and then orally for 3 weeks in female C57BL/6 mice with chromium-induced allergic contact dermatitis [79]. The anti-inflammasome activity of pterostilbene in hyperuricemia model has been demonstrated by an in vitro assay where NLRP3 inflammasome and epithelial–mesenchymal transition in renal cells have been stimulated by TGF-β [80]. The cells treatment with 2 µM pterostilbene caused a reduction in NLRP3 inflammasome by inducing autophagy, a cellular process that is activated when cellular stress events, such as inflammatory factors release, occur [140]. Hyperuricemia disease has also been considered to demonstrate the anti-inflammasome activity of the stilbenoid glucoside polydatin present in grape juice. In the considered in vivo model [81], male Sprague Dawley mice with potassium oxidate-induced hyperuricemia and daily treated with oral 25 and 50 mg/kg (2 and 4 mg/kg) polydatin for 7 days have shown a dose-dependent decrease in IL-1β, TNF-α, IL-6, NLRP3, and caspase-1, measured in the kidney tissues, with respect to non-treated mice. Polydatin has also shown to be effective in reducing NLRP3 inflammasome in both in vivo and in vitro dry-eye disease models when used at 0.05/0.5% ocular solution and 0.1/1/10 µM solution, respectively [82]. Again, an eye inflammation event has been simulated for the anti-inflammasome activity study of cyanidin-3-*O*-β-glucoside (C3G), a phenolic molecule mainly present in red–violet fruits. In particular, 4-hydroxyhexenal-induced inflammation in human retinal pigment epithelial cells has shown to be less severe when cells have been pre-treated for 2 h with 50 and 100 µM C3G, showing a dose-dependent decrease in NLRP3, IL-18, IL-β, and caspase-1 [83]. C3G has also been effective in reducing NLRP3 inflammasome activation in an in vivo hepatic inflammation model in male C57BL/6 J mice, where the administration of a 200 mg/kg/day (16 mg/kg HED) C3G dosage for 8 weeks caused a decrease in NLRP3, IL-18, IL-1β, and caspase-1 expression in serum and liver, together with blocking the NF-κB signaling pathway [84]. Green tea is characterized by the presence of several bioactive polyphenols, of which epigallocatechin-3-gallate is the most abundant. This molecule has shown to be very effective in attenuating NLRP3 inflammasome in both in vivo and in vitro lung injury models [85]. In particular, a male Balb/C mice model of acute pancreatitis induced lung injury has been treated with several epigallocatechin-3-gallate dosages (5, 10, 20, 40, and 80 mg/kg) (0.4, 0.8, 1.6, and 3.2 mg/kg HED) for 4 weeks, and a notably dose-dependent decrease in IL-1β inflammation factor has been observed after treatment. Similarly, the in vitro treatment of injured adherent cells with 2.5, 5, and 10 µM epigallocatechin-3-gallate has shown a dose-dependent decrease in caspase-1, IL-1β, and ROS. The same results have been observed in both in vivo and in vitro models of microglial inflammation and neurotoxicity where the use of 2 mg/kg/day (0.16 mg/kg HED) and 10 µM of epigallocatechin-3-gallate has reduced the NLRP3 inflammasome factors expression [86]. Resveratrol has shown to be very effective in reducing NLRP3 inflammasome when encapsulated in poly(lactic-co-glycolic acid) nanoparticles (Res NPs), in both in vitro and in vivo kidney injury models [87]. In particular, kidney cells with LPS/ATP-induced inflammation have shown a dose-dependent reduction in NLRP3, pro-caspase-1, cleaved-caspase-1, and IL-1β expression when treated with 25, 50, and 100 µM Res NPs. The same dose-dependent results have been observed by treating mice injured kidney with 2 and 4 mg/kg 2 times a week for 4 weeks.

#### 3.1.5. Polyphenols Mixtures

Food extracts characterized by the presence of polyphenols mixtures have also shown to be effective in the inhibition of NLRP3 inflammasome. In this case, it is not possible to have an idea about the contribute of single molecules that are present in the mixture, also in consideration of the synergistic, additive, and antagonistic effects that can occur [141]. Anyway, the study of food-derived mixtures is also important, considering that whole foods, food supplements, and nutraceutical products are generally a mixture of several compounds.

Fermented non-digestible fraction (FNDF) of baked corn (*Zea mays* L.) and common bean (*Phaseolus vulgaris* L.) snacks are food products, rich in polyphenols, such as gallic acid and other compounds, such as butyric acid and verbascose, that present an in vitro anti-inflammatory activity. Regarding the NLRP3 inflammasome complex, the FNDF pure components inhibited the NLRP3 assemblage, decreasing caspase-1 activity, IL-1β, and apoptosis in THP-1 cells and differentiated Caco-2 cells after NLRP3 inflammasome activation [88].

Green tea is a plant with well-known antioxidant and anti-inflammatory activities based on the high polyphenol contents, with epigallocatechin-3-gallate (EGCG) being the most abundant one. In a recent work, the green tea polyphenols (GTPs) have reported a modulating activity against the NLRP3 inflammasome activation [89]. In particular, GTPs and EGCG were administered at ICR mice with induced liver damage and the expression levels of NLRP3 inflammasome proteins were determined by Western blot analysis. The results showed a significant down-regulation of NLRP3, ASC, caspase-1, and IL 1β protein expressions, indicating a reduction in NLRP3 signaling in mice.

In another study, the GTPs were administrated to the same mice, in a dose (100–200 mg/kg) (8 and 16 mg/kg HED) comparable with the normal drinking tea levels consumed by humans, in order to determine their protective effects against the inflammasome activation. The results confirmed that GTPs inhibited the NLRP3, ASC, and caspase-1 p20 expression in a dose-dependent manner [90].

Soy isoflavones represent an interesting flavonoid choice for the treatment of many inflammation disorders. A recent study conducted on dextran sodium sulphate (DSS)-treated mice reported the inhibiting capacity of soy isoflavones on NLRP3 inflammasome complex expression, reducing the NLRP3, caspase-1 p20, and ASC protein levels and suppressing the release of IL-1β and IL-18 [91].

Red raspberry polyphenol extracts are rich in anthocyanins, ellagic acid, myricetin, (−)-epicatechin, and (+)-catechin. The extract was administrated to C57BL/6 mice revealing the attenuation of NLRP3 inflammasome activation in adipose tissue macrophages and epididymal white adipose tissue. The downregulation of NLRP3 inflammasome activation, leading to reduced expression levels of IL-1β, IL-18, and NLRP3 proteins, was also observed in vitro on C3H10T1/2 cells [92].

### 3.2. Organosulfur Compounds

Organosulfur compounds (OSCs) are a class of molecules usually present in several food sources, such as cereals, legumes, vegetables, and fruits. However, the main food sources of OSCs belong to the *Allium* (garlic, onion) and *Brassica* (broccoli, cabbage, cauliflower) genera [142] and are responsible for the typical flavor of these matrices. This group, that mainly includes isothiocyanates, indoles, allylic sulfur compounds, and sulfones, is well known to possess several biological activities, such as antioxidant, anticancer, antimicrobial, and anti-inflammatory [143,144]. Among the possible mechanisms considered to be involved in the anti-inflammatory activity, inhibition of NLRP3 activation has been demonstrated to be the action mode of some OSCs. Allicin, an isothiocyanate compound very abundant in garlic, has shown to reduce the acrylamide induced inflammation in both Kupffer and Sprague Dawley rat liver cells by inhibiting several inflammation pathways that activate the NLRP3 inflammasome in liver [93]. In both in vitro (1 mM acrylamide and 3.75, 7.5, 15 μM allicin) and in vivo (30 mg/kg/d acrylamide and 25 or 50 mg/kg/d of allicin) (2 or 4 mg/kg HED) studies, by comparing the cells treated with acrylamide and allicin/acrylamide, a decrease in several inflammation factors involved in the activation of NLRP3 inflammasome was observed in allicin groups. In particular, allicin has shown to reduce the release of ROS and ERS (endoplasmic reticulum stress) factors that represent the activation signals for NLRP3 inflammasome. Another study has been carried out on Kupffer and liver cells in order to observe the effect of benzyl isothiocyanate, an isothiocyanate compound present in cruciferous vegetables, in reducing the inflammation events in diet-induced NASH [94]. When benzyl isothiocyanate was administrated to Kupffer cell cultures (2.5 or 5.0 μM benzyl isothiocyanate) or male C57BL/6 J mice with induced NASH (1 g/kg/d benzyl isothiocyanate for 9 weeks) (80 mg/kg HED), a reduction in NLRP3 inflammasome activation was observed, through a reduction in NLRP3, p20 caspase-1, and IL1-β expression in blood and liver; moreover, benzyl isothiocyanate has shown to reduce the release of cathepsin β, an inflammasome assembler, and the interaction of cathepsin B with NLRP3. Similar to what described in the previous study, sulforaphane, an allylic sulfur molecule typical of broccoli and other cruciferous vegetables, has shown to reduce the expression of NLRP3, p20 caspase-1, and IL1-β in cerulean-induced acute pancreatitis in BALB/c mice, through a 3-day treatment with 5 mg/kg (0.4 mg/kg HED) of sulforaphane [95]. Finally, another OSC typical of *Allium* genus vegetables, methylsulfonylmethane, has shown to have anti-NLRP3 inflammasome activity in in vitro assays on human and mouse macrophages by using different methylsulfonylmethane solution concentrations (0.3, 0.5, 1.0, 2.0, 4.0, and 8.0%) [96]. In particular, the inhibition of NLRP3 inflammasome was demonstrated to occur at several steps, namely by blocking the NF-κB signaling and pro IL1-β expression and by reducing IL-1β production and inhibiting mitochondrial ROS production.

### 3.3. Terpenes and Terpenoids

Terpenes are a class of volatile hydrocarbon compounds derived from two or more isoprene units and represent the main components of vegetable essential oils. In recent years, a paramount interest for these molecules has emerged, since several biological activities, such as antitumoral, anti-inflammatory, antibacterial, antiviral, antimalarial, and cardiovascular modulation have been demonstrated [145]. In particular, among the different proposed anti-inflammatory modes of action, the inhibition of NLRP3 inflammasome has shown to be the typical mechanism of action of these compounds. Carnosic acid, a diterpenoid mainly present in *Rosmarinus* and *Salvia* plants, has been demonstrated to reduce the evolution of DSS-induced colitis in male Balb/c mice daily gavaged with 50 or 100 mg/kg (4 and 8 mg/kg HED) of carnosic acid for 10 days by inhibiting the activation of caspase-2 and the release of pro-inflammatory cytokines and ROS in the colon tissue [97]. Moreover, in this study, the anti-NLRP3 inflammasome activity of carnosic acid has shown to be comparable with those of 5-aminosalicylic acid, a standard of care for the treatment of colitis disease. The anti-NLRP3 inflammasome effect of geranylgeraniol, a diterpenoid naturally occurred in vegetable oils such as flax, sunflower, and olive, has been studied in an in vitro model of programmed cell death induced in Daoy cell lines after treatment with 10 μM statins or mevalonate for 24 h [98]. The addition of 50 μM geranylgeraniol, following the treatment with statins or mevalonate, has shown to be effective in the reduction in cell death by inhibiting the expression of NLRP3 gene after 24 h. Conversely to what observed in the previous studies, in the following work the beneficial effect of a terpene (kaurenoic acid) has been associated with a stimulation of the NLRP3 inflammasome activation. In particular, kaurenoic acid, a diterpenoid found in several natural spices, such as the fruits of *X. aethiopica*, has shown to possess a dose-dependent (at 10, 30, 50, 70, and 90 μM) triggering effect of NLRP3 inflammasome in BALB/c mice macrophages infected with *L. amazonensis* promastigotes by increasing the production of the inflammatory mediators NO and IL-1β [99].

Although not present in natural sources used as foods, other diterpenoids, sesquiterpenes, iridoids, phytocannabinoids, and derivatives typical of plants used in Eastern and Western medicine, namely α-bisabolol, aucubin, abscisic acid (phytohormone), cannabidiol, phytanyl amine, triptolide, tanshinone IIA, sodium tanshinone IIA sulfonate, paclitaxel, phorbol myristate acetate, andrographolide, oridonin, glaucocalyxin A, and teuvincenone F have shown to be effective in the prevention of NLRP3 inflammasome activation by blocking the release of inflammasome enhancers, mainly IL-1β, IL-4, IL-6, IL-12, IL-18, caspase-1, and ROS [146,147,148,149,150,151,152,153]. Similarly, other terpenoids, such as the triterpenoid celastrol [154] and the sesquiterpenoids of *Ainsliaea yunnanensis* [155], have shown to be involved in the modulation of NLRP3.

### 3.4. Fatty Acids

In general, most of the evidence highlighted that SFAs acted as priming signals of inflammasome activation, whereas MUFAs and PUFAs have been shown to impair this activation, but these assumptions have frequently been challenged [31]. To better understand the FA implication in NLRP3 inflammasome, data reported in the literature will be described according two categories: SFAs and UFAs.

#### 3.4.1. Saturated Fatty Acids

Different research groups attempted to investigate the mechanisms underlying SFA-mediated NLRP3 inflammasome activation. Wen et al. demonstrated palmitate acid inhibits AMP-activated protein kinase in LPS-primed bone marrow-derived macrophages followed by the accumulation of mitochondrial ROS, thus activating the NLRP3-ASC inflammasome and causing caspase-1, IL-1β, and IL-18 production [16]. According to Robblee et al., palmitate and stearate acids could induce activation of one of the three endoplasmic reticulum stress sensors, IRE1α (inositol-requiring enzyme 1-α), through the saturated phosphatidylcholine accumulation, and mediate the NLRP3 inflammasome activation in LPS-primed bone marrow-derived dendritic cells [100]. More recently, Gianfrancesco et al. suggested that the accumulation of saturated phosphatidylcholine induced by SFAs led to the loss of membrane fluidity and the disruption of Na^+^, K^+^-ATPase transmembrane protein, resulting in an increase in K^+^ efflux, which is considered a NLRP3 activator [156].

However, few studies reported the anti-inflammatory effects displayed by different SFAs. As an example, virgin coconut oil (VOC), mainly composed of medium chain saturated fatty acids (6–12 carbons), reduced IL-1β protein, caspase-1, and NLRP3 genes expression at different doses in AD (receiving Amyloid-β) and in high-fat diet (HFD) models both in vitro and in vivo [101]. The anti-inflammatory properties of VOC might be ascribed to lauric acid (which represents up to 55% of the total) that is considered the precursor of monolaurin, which has been shown to modulate immune cell proliferation and, through this, it can control inflammasome [157].

#### 3.4.2. Unsaturated Fatty Acids

In the literature, most of the studies on NLRP3 inflammasome and fatty acids are focused on PUFAs. In particular, fish oil, a matrix particularly enriched in ω-3 DHA and EPA, has been extensively studied. All data reported the anti-inflammatory properties of EPA and DHA resulting in a downregulation of NLRP3 gene expression and in a decreased secretion of pro-inflammatory cytokines (IL-1β and IL-18) in obesity models [102,103], in DSS-induced colitis in mice [104], in LPS-induced Kupffer cells [105], and in both prefrontal cortex and hippocampus of rats [106]. Nevertheless, numerous studies have explored the anti-inflammatory mechanisms of PUFAs. Dang et al. suggested fish oil could attenuate the LPS-induced neuroinflammation and oxidative through modulation of P2X7R/NLRP3 inflammasome axis [106]. Miao et al. underlined that the mechanisms involve inhibition of ROS production, mediation of NLRP3/ASC/caspase-1 signaling pathway, regulation of gut microbiota, and SCFAs levels [104]. More recently, Quingyao et al. assumed that DHA-mediated NLRP3 inflammasome inhibition was due to the blockade of high glucose-induced TXNIP via the PI3K/Akt pathway in pre-adipocytes [103]. Other mechanisms through which ω-3 PUFAs reduce metabolic inflammation may include the G protein-coupled receptor 120 (GPR120) and GPR40, which interact with NLRP3 and inhibited the NLRP3 inflammasome complex assembly [42,105].

Interestingly, different properties have been reported between ω-3 and ω-6 PUFAs. Schuster et al. investigated the anti-inflammatory activity of different fatty acids (SFAs, MUFAs and PUFAs) in macrophages, blood monocytes, and hepatocytes, confirming the data described above. Nevertheless, when compared with ω-6 PUFAs, ω-3 were more potent in inhibiting ATP-mediated NLRP3 inflammasome [158]. Conversely, Yan et al. found out that ω-6 PUFAs failed to block IL-1β secretion induced by nigericin [42]. All these findings suggest that fatty acids should be considered individually in terms of defining potential differences with respect to metabolic or inflammatory properties, or both.

### 3.5. Carotenoids

Recently, only two carotenoids were studied in vivo for the modulation of NLPR3 inflammasome. Zeaxanthin dipalmitate, a lipophilic antioxidant whose primary presence in some functional fruits (e.g., *Lycium barbarum*) has been confirmed, demonstrated the ability to counteract ethanol-induced hepatic damage in a murine model of AFLD targeting the converged AMPK-FoxO3a mitophagy and NPLR3 pathway combined to P2X7 and adiponectin receptor 1 on the hepatocyte membrane [159]. Since the role of NLRP3 in virtually all liver diseases has been demonstrated, this compound or foods containing it can have a positive impact or a preventive effect against a large range of human pathologies. Conversely, astaxanthin, which is poorly represented in typical Western diet but is rich in seafood, exerted an indirect NPLR3 modulation (−23%) via the modification of the gut microbiota in a murine model of inflammation and metabolic homeostasis when administered as a supplementation (0.04% *w*/*w*) to the normal diet [160].

### 3.6. Proteins and Amino Acid Derivatives

Proteins, peptides, and amino acids appear to be involved in the inhibition of the inflammasome pathway. Two short peptides, RDP2, RDP3 (rice-derived-peptide-2, AAAAGAMPK-NH2, 785,97 Da), and RDP3 (rice-derived-peptide-3, AAAAMAGPK-NH2, 785,97 Da), were identified and isolated from the aqueous extract of the shelled fruits of *Oryza sativa* and studied to verify the antigout effects [107,108]. The RDP2 peptide [107] study was conducted on hyperuremic mice, that were injected intraperitoneally once a day for 7 days, and divided into 7 groups: control, model, allopurinol (Allo, 10 mg/kg, 0.8 mg/kg HED), benzbromarone (Benz, 8 mg/kg, 0.6 mg/kg HED), and 3 RDP2 groups (5, 10, and 100 μg/kg, 0.4, 0.8, and 8 μg/kg HED). The RDP2 groups induced a reduction in serum uric acid levels by decreasing renal inflammation. Indeed, the content of serum IL-1β, the production of which depended on NLRP3 inflammasome, was significantly decreased in hyperuricemic mice treated with the RDP2 peptide, and the expression of NLRP3, ASC, and caspase-1 in the kidneys was reduced. A model of hyperuremic mice was established to explore the mechanism and function of the RDP3 peptide [108] and animal tests were performed by dividing mice into various groups: control, model, allopurinol, benzbromarone, and RDP3. Mice were injected with uric acid intraperitoneally to induce hyperuricemia; then, for 7 days, the groups were treated with intraperitoneal injection of standard drugs allopurinol (10 mg/kg, 0.8 mg/kg HED) or with benzbromarone (8 mg/kg, 0.6 mg/kg HED); instead, the RDP3 groups were treated with intraperitoneal injection of different doses of RDP3 (100 μg/kg, 500 μg/kg and 1 mg/kg, 8 μg/kg, 40 μg/kg, and 0.08 mg/kg). The results showed that serum uric acid concentrations in the RDP3 group were significantly lower than in the other treatments. Furthermore, Western blot analysis was performed to detect NLRP3 inflammasome expression in the kidneys of mice and it was observed that NLRP3 contents in the model group kidneys were higher than those of the allopurinol group, suggesting that NLRP3 was activated. Contrariwise, the expression of the NLRP3 inflammasome was significantly decreased in the RDP3 group, demonstrating that RDP3 reduced inflammation by inhibiting the expression of the NLRP3 inflammasome.

Regarding the hyperuricemia and its associated kidney inflammation, the effects and biological mechanisms of tuna meat oligopeptides (TMOP) were investigated in mice [109]. The conducted experiments indicated that TMOP relieved hyperuricemia dose-dependently and regulated uric acid metabolism in mice with diet-induced hyperuricemia. The administration of TMOP inhibited the activation of the NLRP3 inflammasome complex, and intestinal microbiota-mediated beneficial effects of TMOP were explored by fecal microbiota transplantation.

Concerning the intestinal diseases, the ability of an isoflavone-free soy protein concentrate (SPC) to prevent inflammation and loss of intestinal barrier function have been examined [110]. The cytoprotective effects of soy protein concentrate were analyzed in vitro, and its anti-inflammatory effects in the mouse model of acute ulcerative colitis treated with DSS. In particular, the experiments were conducted on mice fed in the basic diet with the components present in the SPC powder for 7 days a randomized based on weight into control (water and AIN93G diet), DSS (1.5% DSS in drinking fluid and AIN93G), DS6 (1.5% DSS and 6% dietary SPC), and DS12 (1.5% DSS and 12% dietary SPC). SPC mice had lower NLRP3 mRNA levels than DSS-treated control mice, demonstrating that SPC is able to prevent increased pro-inflammatory signaling and thereby moderate colitis severity. Overall, the findings support the efficacy of dietary SPC as a means of preventing colonic inflammation and loss of gut barrier function.

Otherwise, a gluten-derived peptide with high content of glutamine and proline residues, α-gliadin 31–43, has been identified, which appears to induce an innate immune response in the gut [111]. Mice intestinal samples, treated for 4 or 16 h with 200 μL of p31–43 using a curved oral gavage needle, were collected for mRNA evaluation and for histological analysis. The results showed that p31–43 gliadin has an inherent propensity to form oligomers that activate NLRP3 inflammasome and that this pathway is required for intestinal inflammation and pathology when p31–43 is administered orally to mice.

By considering that the redox state of the cell can mediate the production of placental cytokines, which are responsible for the function and development of the placenta and that the NLRP3 inflammasome represents the first line of innate immunity [161], the NLRP3 placental inflammasome was investigated in a study regarding the integration of N-acetyl-cysteine (NAC) in the late gestational diet of sows [112]. A total of 16 sows at day 85 of gestation were selected based on body weight and assigned into 2 groups: the control group (7 sows) and NAC group (9 sows; the basal diet supplemented with 500 mg/kg NAC, 40 mg/kg HED). The effects of NAC on placental redox status, function, inflammasome, and fecal microbiota in sows were explored to elucidate the relationship between the fecal microbiota and the placenta. The results obtained showed that NAC significantly reduced maternal and placental inflammatory cytokines through inhibition of the NLRP3 inflammasome in sows during late pregnancy; suppression of oxidative stress and inflammatory response in sows and placenta can therefore reduce fetal exposure to inflammatory mediators and improve fetal growth.

NLRP3 inflammasome has been reported to play an essential role in the inflammation responses during acute lung injury [162]. Regarding this point, glycine supplementation in lipopolysaccharide-induced acute lung injury in mice was investigated [113]. Indeed, after being treated with aerosolized glycine (1000 mg in 5 mL of 0.9% saline) or vehicle (0.9% saline) once daily for 7 continuous days, the male mice were exposed to aerosolized lipopolysaccharide to induced lung injury in mice. The glycine prevents mucin reduction and upregulation of pro-inflammatory cytokines in lung tissue; this beneficial effect of glycine was associated with modulation of the NLRP3 inflammasome and NRF2 signaling. Carnosine (β-alanyl-histidine) and L-homocarnosine (γ-aminobutyryl-histidine) are the major endogenous constituents of excitable tissues—the brain and skeletal muscles [163]. Their supplementation was also studied in terms of modulation of NLRP3. The neuroprotective and anti-aging carnosine was shown to improve cognitive dysfunction in SAMP8 mice after a 6-week oral administration (100–200 mg/Kg/day) via NLRP3 inactivation and amelioration of the oxidative stress [114]. The effects of L-Homocarnosine against inflammation induced by cerebral ischemia in male albino rats were also analyzed [115]. In this study rats were grouped into control, middle cerebral artery occlusion (MCAO), 0.5 mM L-Homocarnosine + MCAO, and 1 mM L-Homocarnosine + MCAO treatment groups and treated for 45 days. NLRP3 inflammasome levels were substantially elevated in MCAO rats, whereas supplementation with 1 mM L-homocarnosine significantly reduced NLRP3 inflammasome levels to near normal levels.

Since cholinergic degeneration plays a major role in the pathophysiology of AD, it has been investigated whether choline supplementation during adulthood delays the progression of this disease [116]. The study was conducted on the APP/PS1 model of double transgenic mice expressing a chimeric mouse/human amyloid precursor protein (Mo/HuAPP695swe) and a mutant human presenilin 1 (PS1-dE9), both directed to CNS neurons; APP/PS1 transgenic mice were divided into a control group (1.10 g choline chloride per kg, 0.09 mg/kg) and a group supplemented with choline (4.95 g choline chloride kg, 0.4 mg/kg HED). Firstly, it was shown that choline supplementation improves the cognitive and non-cognitive behavioral effects of AD; furthermore, from the results, both an increase in the formation of the synaptic membrane and the inhibition of the NLRP3 inflammasome were observed. Inhibition of inflammasome activation explained the reduced Aβ deposition, microgliosis, and pro-inflammatory cytokine production observed in the brains of APP/PS1 mice after 9 months of choline supplementation.

### 3.7. Saponins and Sterols

Ginseng is considered as a highly valued herb and widely used in dietary supplements and herbal medicines, as it exerts a regulation on endocrine system, nerves, metabolism, and other physiological functions [164,165]. The effectiveness of ginseng is attributable to its main active components, ginsenosides, a class of triterpene saponins with a steroid structure.

It has been confirmed that 25-OCH_3_-PPD (20S,25-methoxyldammarane-3β,12β,20-triol), a ginsenoside isolated from *Panax ginseng*, relieves liver damage, by inducing apoptosis of activated hepatic stellate cells [166]. Considering the correlation between inflammation and liver fibrosis, a study evaluated the regulation of 25-OCH_3_-PPD (5, 10, or 20 mg/kg for 5 weeks, 0.4, 0.8, or 1.6 mg/kg HED) against hepatic fibrosis and inflammation in thioacetamide (TAA)-stimulated mice by activating the LXRs (liver X receptors) pathway [117]. Study results demonstrated that 25-OCH_3_-PPD has a hepatoprotective effect against liver fibrosis and reduces inflammation by regulating P2X7R-mediated NLRP3 inflammasome.

Wang et al. [118] analyzed the effects of saponins isolated from *Panax notoginseng* in ameliorating NLFD through inhibition of inflammasome activation. The saponin extract, compared with conventional red ginseng, contained significantly increased amounts of ginsenosides Rh1 (10.34-fold) and Rg2 (7.1-fold), which are the main components highlighting the pharmacological activity of ginseng. In this study mice were fed a fast-food diet for 16 weeks to induce NLFD and then treated with saponin extract (50 or 150 mg/kg) for 9 weeks to determine the effects of the saponin. Particularly, it has been observed that Rh1 and Rg2 ginsenosides exerted anti-inflammatory effects and inhibited NLRP3 inflammasome by promoting mitophagy and alleviating mtROS production.

The characteristics of the ginsenoside compound K (CK) are peculiar, a final metabolite of panaxadiol ginsenosides. CK seems to be involved in the stimulation of insulin secretion and its protective effect against diabetic nephropathy has been elucidated in inhibiting the oxidative stress, NLRP3 inflammasome, and NF-κB/p38 [167,168].

Numerous studies have demonstrated the neuroprotective action and antidiabetic properties of CK, indicating its potential in the treatment of memory disorders and cognitive dysfunctions related to diabetes mellitus; in particular, a study tried to assess the effects of CK against memory impairment and cognitive dysfunction in diabetic db/db mice treated with 10 mg/kg of CK for 12 weeks [119]. The results showed that the CK treatment improved insulin resistance, alleviated cognitive dysfunctions, relieved oxidative stress, attenuated inflammatory responses in the hippocampus, and inhibited NLRP3 activation. Specifically, CK downregulates inflammatory cytokines and mediator production by suppressing the NLRP3 inflammasome pathway.

Magnesium isoglycyrrhizinate, a magnesium salt of 18α-glycyrrhizic acid stereoisomer extracted from the roots of the plant *Glycyrrhiza glabra*, acts as a hepatoprotective agent in the immune and anti-inflammatory modulation of liver disease [169]. The effects of magnesium isoglycyrrhizinate on the characteristics of the metabolic syndrome in fructose fed rats were investigated [170]. Rats were given 100 mL of water containing 10% fructose for 6 weeks, followed by treatment with saline injection, 10, 20, and 40 mg/kg (0.8, 1.6, and 3.2 mg/kg HED) of magnesium isoglycyrrhizinate (by intraperitoneal injection) or 4 mg/kg (0.3 mg/kg HED) of pioglitazone (for intragastric administration) for an additional 11 weeks. The data showed that magnesium isoglycyrrhizinate inhibited the activation of the NF-κB/NLRP3 and thus reduced the immunological–inflammatory response, preventing hepatic lipid metabolic disorder and accumulation in high fructose conditions [120].

Physalin B, one of the main active withanolides existing in *Physalis alkekengi* L. var. *franchetii*, displayed anti-inflammatory activity in intestinal ischemia [171]. The Physalin B antiulcer effects in mice with ulcerative colitis DSS-induced were evaluated [121]; mice were injected intraperitoneally with Physalin B (250 μL) once daily for 7 days. Body weight, colonic length, disease activity index, pathological changes in colonic tissue, cytokine levels, NF-κB pathway, protein levels of related pathways, and NLRP3 activation were measured. The results of this study provided evidence that Physalin B could significantly inhibit the production and secretion of various inflammatory factors: Physalin B reduced the pro-inflammatory cytokine levels of mice with colitis, suppressed the NF-κB cascade, STAT3-arrestin1 signaling, and inhibited NLRP3 inflammasome activation.

A plant phytosterol isolated from *Moringa oleifera*, β-sitosterol, was studied to evaluate its anti-inflammatory activity in two cell lines [122]. Tween 80 surfactant was used to produce a dispersion of small particles of β-sitosterol dissolved in dimethyl sulfoxide and applied to macrophages to evaluate the anti-inflammatory activity of β-sitosterol. Cells were incubated with BSS (7.5–60 μM). An increase in the solubility of water-insoluble phytosterols has been reported thanks to the formation of nanoparticles, the formulation of which is able to inhibit the signal transduction pathways of inflammation in macrophages. The results indicated that β-sitosterol dispersed well in the medium as nanoparticles, suppressed the secretion of inflammatory factors from keratinocytes and macrophages induced by PGN, TNF-α, or LPS (such as TNF-α, IL-1β, IL-6, IL-8, and ROS), significantly reduced NLRP3 expression and inhibited caspase-1 activation.

### 3.8. Polysaccharides

Polysaccharides from different sources (mushroom, herbs, plant, seaweed) have proved to exert an anti-inflammatory role via affecting the NLRP3 inflammasome pathway. However, a detailed mechanistic study on how polysaccharides modulate NLRP3 activation remained to be explored. In particular, several polysaccharides involved in the modulation of NLRP3 inflammasome belong to traditional Chinese medicine, but they are also present in dietary plants. As an example, the major bioactive component of *Trametes orientalis*, used for the treatment of pulmonary disease, is a polysaccharide composed of galactose, glucose, mannose, and arabinose with molar ratios of 5.79:5.77:3.45:1.20 (average MW 63000), which proved to inhibit the activation of NLRP3 inflammasome and the release of pro-inflammatory cytokines, including TNF-α, IL-1β, and IL-6, by the detection of expression levels of proteins in lung tissue involved in the NLRP3 inflammatory pathway [123]. Liang et al. found out the anti-inflammatory activity of polysaccharides extracted from *Dendrobium officinale* (DOPS) was likely to via suppressing mRNA expression of NLRP3, ASC, caspase-1, IL-1β, and IL-18 both in vivo, in DSS-induced acute ulcerative colitis mice, and in vitro, in LPS-stimulated NCM460 cells model, through a possible downregulation of β-arrestin1 expression (which could positively regulate NLRP3) [124]. A recent work by Li et al. reported CYP-1, a newly characterized mannoglucan from Chinese yam was able to suppress the expression of several key genes involved in colonic inflammatory signaling pathways (such as NF-κB and NLRP3) in DDS-induced colitis mice [125]. *Ganoderma lucidum* polysaccharides (GLPS), isolated from a Chinese medicinal mushroom, proved to markedly inhibit liver inflammatory factors via suppression of NLRP3 in liver tissue. Indeed, GPLS-treated acute liver injury mice exhibited a significantly decreased protein expression levels of NLRP3, ASC, and caspase-1 [126]. *Armillariella tabescens* (AT), that belongs to the family of *Tricholomataceae*, is a well-known traditional medicinal mushroom, characterized by 86.52% of polysaccharides (mannose, arabinose, and fucose at a molar ratio of 1.6:1.0:2.7.). AT possesses potent antioxidant and anti-inflammatory properties by possibly involving the repression of the TXNIP/NLRP3 inflammasome pathway in the liver of T2D mice [127]. Among the plant-derived polysaccharides, low methoxyl pectin (LMP) showed an important role in the autoimmune diabetes through suppression of NLRP3 and associated proteins expression in cecum (NLRP3, caspase-1-p20, cleaved IL-1𝛽, and cleaved IL-18) [128]. The NLRP3 inhibition could be ascribed to the increase in the SCFAs (short chain fatty acids) by gut microbiota, induced by LMP supplementation, which act as histone-deacetylase (HDAC) inhibitors towards the NLRP3 inflammasome. Furthermore, Wu et al. pointed out that LMP supplementation also suppressed NLRP3 activation in pancreas, but a detailed mechanistic study on how LMP modulates NLRP3 inflammasome activation in pancreas has not yet been performed [129].

Recently, Castro-Alves et al. investigated the activity of non-digestible carbohydrates (NDCs) from chayote fruit, which consist mainly of pectic homogalacturonan and highly branched rhamnogalacturonan-II, as well as hemicellulosic material including glucomannan, xyloglucan, and glucurono(arabino)xylan, in human THP-1 macrophage-like cells. Results showed that NDCs indirectly inhibit NLRP3 activation through the interaction between the NDCs and other pattern-recognition receptors that are essential to induce priming signals required for NLRP3 activation [130].

Several papers highlighted the anti-inflammatory activity of different polysaccharides probably ascribed to NLRP3 inhibition by decreased protein levels of NLRP3, ASC, caspase-1, and IL-1β [172,173,174]; however, few works assessed the pro-inflammatory efficacy of some polysaccharides towards the NLRP3 inflammasome. In particular, Liu et al. found out that the polysaccharide SF-2, a mannoglucan sulfate isolated from starfish (*A. rollestoni*), could improve the release of cytokines and the expression of NLRP3 in primary macrophages, confirmed by the elevated expression of NLRP3, cleaved caspase-1, and ASC proteins [131].

### 3.9. Vitamins and Derivatives

The importance of lipophilic vitamin E and its derivatives, which mainly occur as abundant nutrients in oily nuts and seeds, in inflammation and oxidation processes has been elegantly reviewed by Wallert and collaborators [175]. Different forms of vitamin E or their long-chain metabolites can interfere with ROS production, NF-kB priming, or NLRP3 activation. Recently, further studies confirmed these results in two different murine models of HFD and alloxan-induced diabetes, especially regarding γ-tocopherol supplementation, as such, or in more complex extracts (e.g., Rosa mosqueta oil contains about 74 g/100 g oil of α-tocopherol and 359 g/100 g oil of γ-tocopherol, stripped corn oil) [176,177].

Vitamin D and its derivatives were also studied, alone or in association with pro-biotics and microelements as food supplements, for their effects on the modulation of inflammasome halting the priming step required for NLRP3 (and NLRP1) activation in immune cells, in placental explants from preeclamptic and normotensive pregnant women, in clinical trials involving COVID-19 patients [178], in the proliferative diabetic retinopathy pathogenesis, in diabetic corneal wound healing and reinnervation, in non-alcoholic fatty liver disease, and in acute kidney injury.

### 3.10. Probiotics, Symbiotics, and Their Main Components

Lactic acid bacteria (LAB) are currently used as food supplements for human health as probiotics due to their modulatory effects on intestinal conditions, immunomodulatory function, and host metabolism. The effect of each probiotic is dependent on the cell line and species used. To test the anti-inflammatory action of LAB or their main components (e.g., butyrate at 200 mg/kg, 16.3 mg/kg HED) via the inhibition of inflammasome activation, *Lactobacillus paracasei* KW3110 (1 × 10^6^ cells/mL), *Bifidobacterium infantis* (2 × 10^8^ CFU/mL) in combination with the prebiotic xylooligosaccharide (XOS, 230 mg/kg, 18.7 mg/kg HED), *Enterococcus faecium* NCIMB 10415 (1 × 10^7^ CFU/mL), and heat-killed cells of *Enterococcus faecalis* (17 mg/kg, 1.4 mg/kg HED) were recently proposed in different in vitro and in vivo disease models of DSS-induced ulcerative colitis [179], HFD-induced T2D [180], cerulein-induced acute pancreatitis [181], and colitis-associated colorectal cancer [182], as well as on porcine dendritic cells [183]. The beneficial effects of a direct modulation of NLRP3 were also confirmed by a downregulation of pro-inflammatory cytokines, upregulation of anti-inflammatory players. These results have been also described feeding animals or treating cells derived from livestock with probiotics (1 × 10^5^ CFU of *Lactobacillus rhamnosus* GR-1 and 3 × 10^5^–2 × 10^7^ CFU of *Lactobacillus johnsonii* L531) with the aim of limiting the inflammatory damage of bacterial infections induced by *Escherichia coli* in porcine mammary epithelial cells, MAC-T cells and in a mouse mastitis model, or *Salmonella typhimurium* in IPEC-J2 cells [184,185,186]. The administration of these probiotics allowed limited bacterial adhesion to cells and bacterial alteration of cellular morphology, thus removing the first cause of ROS production and immune system activation. The NLRP3 attenuation downregulated ILs, TNF-α, and chemokine Cxcl2 expression and, concurrently, decreased the expression of autophagic receptor SQSTM1/p62 and induced tight junction injury.

## 4. Food and Nutraceuticals Components Bioavailability and Toxicity

Apart from table sugar, foods are “complex matrices” made up of different components, divided into macronutrients (proteins, carbohydrates, and fats), micronutrients (vitamins and minerals), and water. The presence of other minority components also increases the complexity of food matrices. Some of them are defined as “bioactive compounds” because, based on scientific evidence, it is assumed that they can have a positive effect on our organism and can play a fundamental and important role in modulating inflammasomes [12,30,35].

The “bioactive compounds” belong to many different categories based on their chemical nature and on the type of positive effect they could exert on human health. Commonly, they are not considered nutrients and are referred as “essential and non-essential compounds” that occur in nature, are part of the food chain, and can help to protect ourselves from illnesses and improve our quality life [1,187]. Although the most known and studied “bioactive compounds” are of plant origin, it is wrong to think that they are not present in foods of animal origin. Conjugated linoleic acid, taurine, carnitine, carnosine, glutathione, and lipoic acid are some examples, together with bioactive peptides, which confirm the importance of all food types in our diet [188,189].

Certainly, the most numerous classes of “bioactive compounds” are represented by phytochemicals: molecules of various kinds such as carotenoids, tocopherols, phytosterols, phenolic compounds, etc., but, in any case, these are of vegetable origin. Among all these different metabolites that are important for their health benefits, the phenolic compounds have been studied most extensively [190,191]. These constituents, with more than 5000 structurally diverse molecules of various types, are the most numerous, abundant, and widely distributed “bioactive compounds”, very important for their potential beneficial effects, which are also associated with inflammasome disorder in diseases [1,17,53,190,192]. Many of these positive effects on safety and efficacy have been studied in vitro and on animal models and have yet to be confirmed with absolute certainty on humans with clinical trials [193,194,195]. It is well known that the “bioactive compounds”, whatever their origin (from animals or plants source), due to the food complexity and in order to exert their beneficial health action, need to be released from the food matrix after ingestion, and then metabolized [196]. As different researchers highlighted, the global food matrix composition, with the presence of nutrient interaction, has a crucial role on the health benefit of “bioactive compounds”; however, some other specific characteristics can determine the biological activity of these metabolites [17,197]. The factors that influence the absorption and bioavailability of “bioactive compounds” are numerous, one of these is represented by the cell wall structure and properties, since, often, they are resistant to degradation in the upper gut [196]. In addition, the in vivo biological activity of many high molecular weight compounds, such as flavonoids, is influenced considerably by molecular structure, binding to other molecules (i.e., by esterification or glycosylation), different isomeric configuration and their interaction with macromolecules, such as proteins and dietary fibers [196,198,199,200,201]. Among these factors, we must include different transport and diffusion mechanisms of ingested food “bioactive compounds” because their bioavailability can be blocked and/or modified significantly by certain nutrients [202]. Moreover, as many drugs, also bioactive food compounds are subjected to several metabolic and enzymatic processes that modify the structure and could be responsible for molecular forms, which are different from the original constituent of the ingested food and can show a different effect (increase, decrease, or toxic) in presence of specific nutrients (i.e., lipids) [196].

The intake of “bioactive compounds” in the human diet varies enormously in relation to the type, quantity, and quality of foods consumed. Generally, the most important food sources of bioactive nutrients are vegetable, fruits, chocolate, tea, wine, olives, and spices; however, their concentration in the same food varies, often significantly, in relation to the cultivation techniques and food processing, the degree of ripeness, and the time elapsed between harvesting and consumption [198,203]. Thus, because different foods contain a huge variety of bioactive compounds and a lot of them often are poorly absorbed in our intestine, there is great difficulty in establishing the “effective dose”, which is the amount of this substance that we must take to have the expected effect. Furthermore, the “bioactive compounds” stability and, consequently, their antioxidant, anti-inflammatory, and immunomodulatory properties, may be lost during digestion [1,190].

Usually, to improve the sensory attributes and make a product more attractive for taste, consistency, and appearance, many foods are submitted to culinary treatments. Different domestic practice and cooking techniques (i.e., boiling, roasting, stir-frying, microwaving, and streaming) have been compared to define the optimal process to reduce the degradation of the “bioactive compounds”. It was observed that food nature, cooking time, temperature, addition of acidulant, spices, or other condiments might influence both the amount of water-soluble “bioactive compounds” and of those fixed to the lipidic matrix [204]. Each food contains a characteristic composition, and the chemical structure of each bioactive metabolite strongly influences the loss extent of individual component. Generally, food processing and even storage frequently cause losses in phenolic compounds [205]. Overall, high-temperature food processing (i.e., roasting, blanching, drying, and pasteurization) considerably reduces the concentration of phenolic compounds in foods. To ensure the greatest phenolic amounts, it is recommended to consume fresh food or use steam cooking with almost no osmotic processes. Other methods, such as microwave cooking, that imply low water and short cooking time, are more recommended to maintain high levels of these compounds inside foods. In fact, when subject to boiling (commonly used for vegetable) or too high temperatures, foods suffer considerable losses of most phenolic compounds, especially flavonol glucosides [204,205,206]. For other bioactive compounds that are part of lipidic membrane or oil fraction of foods (i.e., carotenoids and tocopherols), cooking methods involving water are less aggressive than those using oils for stir-frying [204]. Innovative non-thermal procedures (UV, high hydrostatic pressure, and pulsed electric fields treatments) do not significantly deteriorate bioactive compounds, preserve their antioxidant activity, and provide promising alternative to thermal processing commonly used for food disinfection or to inactivate microbes and enzymes in foods [206].

Although there is evidence showing the beneficial effects of “bioactive compounds” present in foods, more studies suggest that their health benefits instead depend on the synergy and interactions among different molecules [207]. Therefore, in drafting the anti-inflammatory diet it must be considered that bioactive nutrients cannot be from a single food but rather it is the result from the synergy between foods that provide different antioxidant molecules to counteract the inflammatory processes that occur. In addition, as stated above, it is not only important to know how much a nutrient is present in a specific food, but it is even more important to know how much of it is bioavailable.

Despite the growing amounts of studies, definitive conclusions supporting the bioavailability of most “bioactive compounds” from a wide variety of food for optimal nutrition and health well-being are difficult to obtain. Consumers’ demand for well-being pushes towards an increasing request for food supplements and innovative new market segments have been developed to meet their needs. Alternatively, dietary supplements, also differentiated into nutraceutical or functional foods, can be supplied to consumers to deliver a specific bioactive compound or a group of them [1,189]. Generally, nutraceuticals are bioactive compounds derived from food sources. They can include one or more substances that are purported to provide extra health benefits and are considered as a pharmaceutical alternative [208]. They are supplied to consumers in a concentrated form and the ingredients are in higher dose than in normal foods. Therefore, as a drug, the food supplement should be used as a necessary complement in diets that are poor in nutrients and foods, in order to provide more energy and nutrients to our body to properly support its physiological processes. On the other hand, functional foods are foods fortified with an extensive array of bioactive compounds which provide a clinically proven health benefit, beyond their natural properties, when consumed in a regular and consistent manner through diet [189,209]. Therefore, as a drug, nutritional supplementation of nutrients in order to increase the functionality of the immune system has no indication in case of adequate intake; on the contrary, it can be even harmful if it involves exceeding the maximum daily doses.

Nowadays, a great variety of nutraceuticals and functional foods are provided to the consumers; however, their use is largely unregulated, or they are treated differently according to local jurisdictions. Generally, they are more the subject of marketing hype, and for many of these “supplements”, it is not even yet known whether they provide more benefits than risks to consumers. Currently the term “bioactive compound” is not defined in the European regulations; however, the European Food Safety Authority (EFSA) is carrying out scientific evaluations to assess the safety and the toxicity of “bioactive compounds” that can be found in foods [210]. Due to the great number of compounds that can have a potentially positive effect on health, the European Commission has not produced a list of authorized ones, but it has tried to put order for “borderline” products, by identifying the criteria to define a dietary supplement. At present, among food supplement phytochemicals, EFSA claims for hydroxytyrosol, its derivatives (i.e., oleuropein complex and tyrosol), and olive oil polyphenols as substances able to protect the blood lipids from the harmful effects of oxidative stress [211]. With this background, we highlighted that the “bioactive compounds” present in foods can possess a potential therapeutic effect on different diseases associated with the activation of inflammasomes. Anyhow, due to different food matrix effects and compositions, the outcome obtained from concentrations used in in vitro tests are often not achievable through a targeted diet. Currently there is insufficient information available on the safety of supplements or functional foods. They cannot be considered as the emergency remedy and are not intended as a substitute for a varied and balanced diet and a correct lifestyle.

Finally, food consumed as such, or after cooking processes, as well as water, may also contain internal or accidental components with a stimulating effect on NLRP3 inflammasome. Among these, mycotoxins (zearalenone, patulin, deoxynivalenol), trace metals (arsenic, cadmium), environmental and food contaminants (acrolein, 3-monochloropropane-1,2-diol, glycidol, and its esters), and acrylamide are few examples of NLRP3 activators, endowed with harmful inflammatory effects [212,213,214,215,216,217,218,219,220].

## 5. Conclusions

Eating is something we have always done and which, from birth to old age, strongly affects life quality and physical and psychological well-being. Nowadays, consumers are increasingly interested in lifestyles and nutritional habits that can prevent diseases since different studies have evidenced that a relationship occurs between these factors and pathological consequences based on inflammatory processes. In this regard, it should be emphasized that these effects are related, not only to the quantity, but, above all, to the variety of food choices and, in particular, to the inclusion of fruit and vegetables. Plant foods are particularly rich in important bioactive compounds, each of them can produce different effects on the pathway of inflammatory response, confirming the importance of the nutritional pattern (food model) as a whole, rather than the single nutrient or functional compound.

## Figures and Tables

**Table 1:** Summary list of all the discussed food-derived molecules with a NLRP3 inflammasome modulatory activity. Compound name, food source, considered experimental model, dose, treatment duration, and main outcome are described.

Chemical Class	Compound	Food Source	Experimental Model	Dose	Treatment (Duration)	Main Outcome	References
Phenolic acids	Sinapic acid	Vegetables, spices, fruits, cereals, oilseed wine, vinegar	Male Kunming mice (induced colitis)	10, 50 mg/kg	7 days	Dose-dependent reduction in the NLRP3 inflammasome proteins expression.	[57]
	Ferulic acid	Fruits, vegetables, grains, beans, seeds, nuts, grasses, corn, wheat, turmeric	Male Wistar rat (induced kidney injury)	25, 50 mg/kg	15 days	Both doses of ferulic acid administration have produced an amelioration of NLRP3 and caspase-1 proteins expression.	[58]
	Chlorogenic acid	Fruit, coffee beans, eggplants	1. Male and female BALB/c mice (colon tissue)	1. 20, 40 mg/kg	1. 7 days	1. Decrease in NLRP3, ASC, caspase-1 p45, and caspase-1 p20 protein levels.	[59]
			2. RAW264.7 cells	2. Many concentrations of chlorogenic acid	2. Not reported	2. Decrease secretion of IL-1𝛽 and IL-18.	
Flavones and flavonones	Apigenin	Parsley, onions, oranges, tea	1. Male C57BL/6 J mice (induced NAFLD)	1. 50 mg/kg of BW per day	1. 7 days	1., 2.: Reduction in NLRP3, ASC, pro-caspase-1, caspase-1, together with a reduction in ROS production. 3. Reduced NLRP3 protein expression.	1 and 2. [60] 3. [61]
			2. Hepa1–6 cells	2. 16 and 32 μM	2. 24 h		
			3. ISO-HAS human endothelial cells	3. 30 and 50 μM	3. 24 h		
	Isoorientin	Gentiana	1. Male ICR mice (induced hyperuricemia)	1. 5 and 10 mg/kg of BW	1. Single dose	Dose-dependent inhibition of xanthine oxidase activity and interleukin release.	[62]
			2. MXC207 cells	2. 25, 50, 100, 200, and 400 µM	2. 24 h		
	Chrysin	Honey, propolis, carrots, chamomile, fruits, mushrooms	Male Sprague Dawley rats (induced hyperuricemia)	50, 100, and 150 mg/kg of BW	4 weeks	Reduction in IL-1β expression and ROS activity.	[63]
	Luteolin	Chamomile, carrots, olive oil, species	Male Sprague Dawley rats (induced SCII)	50 and 100 mg/kg of BW	14 days	Reduction in NLRP3, IL-1β, and IL-18 expression.	[64]
	Hesperidin methylchalcone	Citrus	Swiss mice (induced gout arthritis)	30 mg/kg of BW	Single dose	Reduction in NLRP3, ASC, pro-caspase-1, and pro-IL-1β mRNA expression.	[65]
	Naringin	Citrus	Male C57BL/6 mice (induced ulcerative colitis)	25, 50, and 100 mg/kg of BW	7 days	Dose-dependent reduction in NLRP3, ASC, caspase-1, and IL-1β expression.	[66]
Flavonols	Quercetin	Fruits, vegetables, seeds, grains	1. Male SPF-Wistar rats (induced liver injury)	1. 100 mg/kg of BW	1. 14 days	1. Reduction in ROS, NF-κB, NLRP3, IL-1β, and IL-18 expression.	1. [67]
			2. ApoE-/- mice (induced atherosclerotic inflammation)	2. 100 mg/kg of BW	2. 16 days	2. Decrease in pro-IL-1β and IL-1β production.	2. [68]
			3. Senescence accelerated mouse P8 and R1	3. 35 and 70 mg/kg of BW	3. 4 weeks	3. Increase in cognitive functions and reduction in NLRP3 activation factors expression.	3. [69]
			4. Caco-2 cell triggered with E.coli	4. 200 µM	4. 12 h	4. Reduction in NLRP3, caspase-1, and IL-1β expression, together with an enhanced ROS scavenger activity.	4. [70]
	Dihydroquercetin	Onions, milk thistle, Douglas fir bark	1. Male C57BL/6 mice (induced liver steatosis)	1. 1, 5, and 25 mg/kg of BW	1. Single dose	Inhibition of NLRP3, caspase-1 cleavage, and IL-1β production.	[71]
			2. Human hepatoma cells HepG2	2. 6.25, 25, and 100 µM	2. 1 h		
	Kaempferol	Tea, vegetables, oranges, wine	Male C57BL/6 mice (induced hepatotoxicity)	30 and 60 mg/kg of BW	7 days	Dose-dependent reduction in IL-1β, TNF-α, and IL-6 expression.	[72]
	Myricetin	Vegetables, fruits, nuts, berries, tea, red wine	Male Wistar rats (induced diabetes)	20 mg/kg of BW	4 weeks	Reduced NLRP3 inflammasome activation.	[73]
Other phenolics	Curcumin	Turmeric	1. Male Sprague Dawley rats (chronic unpredictable mild stress)	1. 100 mg/kg of BW	1. 4 weeks	1. Reduction in IL-1β, IL-6 and TNF-α expression.	1. [74]
			2. Hemodialysis patients	2. 2.5 g of turmeric (95% curcumin) after dialysis	2. 12 weeks	2. Lower expression levels of NLRP3 inflammasome markers (NF-kB, NLRP3 and IL-1β.	2. [75]
	6-shogaol	Ginger roots	1. Human artery smooth muscle cells (induced calcification)	1. Not indicated	1. Not indicated	1. Reduction in NLRP3, caspase-1 and IL-1β expression.	1. [76]
			2. Human THP-1 monocytes	2. 5, 10, 20 and 40 µM	2. 1 h	2. Reduction in the canonical NLRP3 inflammasome-mediated IL-1β secretion.	2. [77]
	Pterostilbene	Grapes, blueberries	1. Female BALB/c mice (induced acute liver failure)	1. 50 mg/kg/12 h of BW	1. 24 h	1., 2.: Decrease in IL-1β, IL-6, caspase-1, TNF-α, and NLRP3 protein expression. 3. Increased autophagy, resulting in a decrease in NLRP3 and caspase-1.	1. [78]
			2. Female C57BL/6 mice (allergic contact dermatitis)	2. 500 mg/kg of BW	2. 5 weeks		2. [79]
			3. NRK-52E cells	3. 2 µM	3. 24, 48, and 72 h		3. [80]
	Polydatin	Grapes juice	1. Male Sprague Dawley rats (induced hyperuricemia)	1. 25 and 50 mg/kg of BW	1. 7 days	1. Dose-dependent decrease in IL-1β, TNF-α, IL-6, NLRP3, and caspase-1.	1. [81] 2 and 3. [82]
			2. Male Wistar rats (induced dry-eye disease)	2. 0.05 and 0.5% ocular solution	2. 4 days	2. Dose-dependent decrease in IL-1β, IFN-α, TNF-α, and IL-6.	
			3. Human conjunctival cell line HCC	3. 0.1, 1 and 10 µM	3. 8 h	3. Dose-dependent decrease in NLRP3 and caspase-1.	
	Cyanidin-3-O-β-glucoside	Red-violet fruits	1. Human retinal pigment epithelium cells, ARPE-19	1. 50 and 100 µM	1. 2 h	1. Dose-dependent decrease in NLRP3, IL-18, IL-β and caspase-1.	1. [83]
			2. Male C57BL/6 J mice (induced hepatic inflammation)	2. 200 mg/kg of BW	2. 8 weeks	2. Decrease in NLRP3, IL-18, IL-1β, and caspase-1 expression, together with the block of NF-κB signaling pathway.	2. [84]
	Epigallocatechin-3-gallate	Green tea	1. Male Balb/C mice (induced acute pancreatitis) 2. Balb/C adherent cells 3. APP/PS1 transgenic mice 4. Mouse microglial cell line BV2	1. 5, 10, 20, 40, and 80 mg/kg of BW 2. 2.5, 5, and 10 µM 3. 2 mg/kg of BW 4. 10 µM	1. 4 weeks 2. 24 h 3. 4 weeks 4. 1 h	1. Dose-dependent decrease in IL-1β. 2. Dose-dependent decrease in caspase-1, IL-1β, and ROS. 3., 4.: Suppressed activation of NLRP3 inflammasome via TLR4/NF-κB pathway.	1. and 2. [85] 3. and 4. [86]
	Resveratrol (encapsulated in poly(lactic-co-glycolic acid) nanoparticles)	Grapes, blueberries, raspberries, mulberries, peanuts	1. Male C57BL/6 mice (induced kidney injury) 2. Epithelial cell line HK-2	1. 2 and 4 mg/kg 2. 25, 50, and 100 µM	1. 2 weeks 2. 24 h	1., 2.: Dose-dependent reduction in NLRP3, pro-caspase-1, cleaved-caspase-1, and IL-1β expression.	1, and 2. [87]
Polyphenols mixture	1. Fermented non-digestible fraction of baked corn and common bean snacks (FNDF) 2. FNDF pure components (include gallic acid)	*Zea mays* L., *Phaseolus vulgaris* L.	Caco-2 cells, THP-1 cells	1. 40, 200, and 300 µg/mL 2. Gallic acid 38.85 µM	48 h	Inhibition of the NLRP3 assemblage, decreasing caspase-1 activity, IL-1β, and apoptosis.	[88]
	1. Green tea polyphenols (GTPs) 2. Epigallocatechin- 3-gallate (EGCG)	Green tea	Male ICR mice (liver tissue)	1. 0.32% GTPs (*w*/*v*) 2. 0.32% EGCG (*w*/*v*)	12 days	Downregulation of NLRP3, ASC, caspase-1, and IL-1β proteins expression.	[89]
	Green tea polyphenols (GTPs)	Green tea	Male ICR mice (liver tissue)	100, 200 mg/kg of BW	7 days	Inhibition of NLRP3, ASC, and caspase-1 (p20) expression in a dose-dependent manner.	[90]
	Soy isoflavones	Soybeans	Male C57BL/6 mice (colon tissue)	50, 100 mg/kg of BW	5 days	Reduction in NLRP3, Caspase-1 p20 and ASC protein levels and suppression of IL-1β and IL-18 secretion.	[91]
	Red raspberry polyphenols	*Rubus idaeus* L.	1. Male C57BL/6 mice (adipose tissue) 2. C3H10T1/2 cells	1. 120 mg/kg of BW per day 2. 10 μg mL^−1^	1. 16 weeks 2. 2 days	1. Attenuation of NLRP3 inflammasome activation in adipose tissue macrophages and epididymal white adipose tissue. 2. Reducing of IL-1β, IL-18, and NLRP3 protein levels expression.	[92]
Organosulfur compounds	Allicin	Garlic	1. Male Sprague Dawley rats (acrylamide treated) 2. Kupffer cells (BNCC341160)	1. 25 and 50 mg/kg of BW 2. 3.75, 7.5, and 15 μM	1. 4 weeks 2. 2 h	1., 2.: Reduced NLRP3 inflammasome activation, decreasing cleaved-caspase-1, IL-1β, IL-18, IL-6, and TNF-α secretion.	1. and 2. [93]
	Benzyl isothiocyanate	Cruciferous vegetables	1. Male C57BL/6 J mice (induced nonalcoholic steatohepatitis) 2. Mice Kupffer cells	1. 1 g/kg of BW 2. 2.5 and 5.0 μM	1. 9 weeks 2. 4 h	1., 2.: Reduction in NLRP3, p20 caspase-1, and IL1-β expression.	1. and 2. [94]
	Sulforaphane	Cruciferous vegetables	BALB/c mice (induced pancreatic injury)	5 mg/kg of BW	3 days	Reduced expression of NLRP3, p20 caspase-1, and IL1-β.	[95]
	Methylsulfonylmethane	Garlic	Bone marrow-derived macrophages	0.3, 0.5, 1.0, 2.0, 4.0, and 8.0%	6 h	Blocking the NF-κB signaling and pro IL1-β expression.	[96]
Terpenes and terpenoids	Carnosic acid	*Rosmarinus* and *Salvia*	Male Balb/c mice (induced acute colitis)	50 or 100 mg/kg of BW	10 days	Reduced release of caspase-2 and ROS.	[97]
	Geranylgeraniol	Flax, sunflower, and olive oils	Daoy cells	50 μM	24 h	Inhibition of NLRP3 gene expression.	[98]
	Kaurenoic Acid	*X. aethiopica* fruits	BALB/c mice macrophages	10, 30, 50, 70, and 90 μM	24 h	Increased the production of NO and IL-1β.	[99]
Fatty acids	Saturated fatty acids	Palmitate acid	LPS-primed bone marrow-derived macrophages generated from wild-type (WT), *Nlrp3*^−/−^, *Pycard*^−/−^, or *Nlrc4*^−/−^	200 or 500 μM	24 h	Inhibition of AMP-activated protein kinase followed by the accumulation of mitochondrial ROS, thus activating the NLRP3-ASC inflammasome and causing caspase-1, IL-1β, and IL-18 increased production.	[16]
		Stearate acid	LPS-primed bone marrow-derived dendritic cells	250 μM	5, 20 h	NLRP3 inflammasome activation mediated by IRE1α activation (inositol-requiring enzyme 1-α) through the saturated phosphatidylcholine accumulation.	[100]
		Virgin coconut oil	Male *Wistar* rats AD (receiving Amyloid-β) and high-fat diet (HFD) models both in vitro and in vivo	8 and 10%	8 weeks	Reduction in IL-1β protein, caspase-1, and NLRP3 genes expression.	[101]
	PUFA	Fish oil	Obesity male *Wistar* rat models	Intragastrically 1 mL/kg per day	8 weeks	Reduction in IL-1β protein, caspase-1, and NLRP3 genes expression.	[102]
		Safflower oil	A. Fat-1 transgenic mice generated onto a C57BL/6 background B. Wild-type mice	10 g safflower oil (per 100 g of diet)	80 days	Caspase-1, IL-1β, and IL-18 reduction. Blockade of high glucose-induced TXNIP via the PI3K/Akt pathway in pre-adipocytes.	[103]
		Walnut oil	DSS-induced colitis in Kunming (KM) male mice	2.5 mL/kg⋅d Walnut oil	27 days	inhibition of ROS production, mediation of NLRP3/ASC/caspase-1 signaling pathway, regulation of gut microbiota and SCFAs levels.	[104]
		DHA	A. ASH-associated mouse LPS-induced Kupffer cell lines B. C57BL/6 J mice	1. 50 μM 2. Intragastrally DHA 50 mg/kg once per day	1. 4 h 2. 7 days	Mechanisms through which ω-3 PUFAs reduce metabolic inflammation may include the G protein-coupled receptor 120 (GPR120) and GPR40 which interact with NLRP3 and inhibited the NLRP3 inflammasome complex assembly.	[105]
		Fish oil	Male Sprague Dawley rats (prefrontal cortex and hippocampus of rats)	1.5 g/kg of BW	3 weeks	Reduced MDA level and mRNA levels of pro-inflammatory cytokines 1 L-1β, IL-6. Mitigation of the LPS-induced P2X7R and NLRP3 activation, downregulated LPS-induced iNOS and NF-κB expression in both prefrontal cortex and hippocampus.	[106]
Proteins and amino acid derivatives	RDP2	*Oryza Sativa*	Kunming mice (induced hyperuremia)	Allopurinolo:10 mg/kg of BW Benzbromaron: 8 mg/kg of BW RDP2: 5, 10, 100 μg/kg of BW	7 days	1. Reduction serum uric acid levels 2. Reduction in NLRP3, ASC, and caspase-1 expression in the kidneys.	[107]
	RDP3	*Oryza Sativa*	Kunming mice (induced hyperuremia)	Allopurinol:10 mg/kg of BWBenzbromaron: 8 mg/kg of BW RDP3: 100 μg/kg, 500 μg/kg and 1 mg/kg of BW	7 days	1. Serum uric acid concentrations in the RDP3 group were significantly lower than in the other treatments. 2. RDP3 reduced inflammation by inhibiting the expression of the NLRP3 inflammasome.	[108]
	TMOP (Tuna meat oligopeptides)	Tuna	ICR mice (induced hyperuremia)	50 mg/kg and 300 mg/kg of BW	8 weeks	Dose-dependent reduction in hyperuricaemia, due to the inhibition of NLRP3 inflammasome complex.	[109]
	Soy protein concentrate (SPC)	Soy	CF-1 mice (induced acute ulcerative colitis)	DSS (1.5% DSS in drinking fluid and AIN93G), DS6 (1.5% DSS and 6% dietary SPC), and DS12 (1.5% DSS and 12% dietary SPC)	7 days	Prevent increased pro-inflammatory signaling and thereby moderate colitis severity.	[110]
	α-gliadin 31–43	Gluten	C57BL/6 mice intestinal samples	200 μl	4/16 h	1. Formation of oligomers that activate the inflammasome.2. Stimulation of IL-1β release.	[111]
	N-acetyl-cysteine (NAC)	Garlic, onions, and leeks	Raised and pregnant sows	500 mg/kg of BW	From day 85 until delivery	Reduction maternal and placental inflammatory cytokines through inhibition of the NLRP3 inflammasome.	[112]
	Glycine	Fish, meat, spirulina algae, soy protein, egg whites	C57BL/6 male mice (induced lung injury)	1000 mg in 5 mL of 0.9% saline	7 days	Prevent mucin reduction and upregulation of pro-inflammatory cytokines.	[113]
	Carnosine	Fish and meat	SAMP8 mice	100–200 mg/kg of BW	6 weeks	Decreased levels of malondialdehyde and reactive oxygen species (ROS), increased activity of superoxide dismutase (SOD) and the level of adenosine triphosphate; NLRP3 inflammasome reduction.	[114]
	L-Homocarnosine	Meat	Ischemic albino Wistar rats	0.5 mM and 1 mM	45 days	Reduction in NLRP3 inflammasome levels to near normal levels.	[115]
	Choline	Eggs, meat, fish, legumes	APP/PS1 transgenic mice	1.10 g/kg and 4.95 g/kg of BW	9 mounths	Reduction Aβ deposition, microgliosis, and pro-inflammatory cytokine production.	[116]
Saponins and sterols	25-OCH_3_-PPD ginsenoside	*Panax ginseng*	C57BL/6 mice (induced TAA)	5, 10, or 20 mg/kg of BW	5 weeks	Reduction inflammation by regulating P2X7R-mediated NLRP-3 inflammasome.	[117]
	Rh1 and Rg2 ginsenosides	*Panax notoginseng*	C57BL/6 mice (induced NAFLD)	50 or 150 mg/kg of BW	9 weeks	Inhibition NLRP3 inflammasome, promoting mitophagy, and reduction mtROS production.	[118]
	Ginsenoside compound K (CK)	*Panax ginseng*	Diabetic db/db and db/m mice	10 mg/kg of BW	12 weeks	Downregulation inflammatory cytokines and mediator production by suppressing the NLRP3 inflammasome pathway.	[119]
	Magnesium isoglycyrrhizinate	*Glycyrrhiza glabra*	Sprague Dawley mice	10, 20, and 40 mg/kg of BW	11 weeks	Inhibition the activation of the NF-κB/inflammasome NLRP3 and reduction the immunological–inflammatory response.	[120]
	Physalin B	*Physalis alkekengi* L.	BALB/c mice	250 μL	7 days	Reduction the pro-inflammatory cytokine levels, suppression the NF-κB cascade and pathway STAT3 and arrestin1 signaling, and inhibition NLRP3 inflammasome activation.	[121]
	β-sitosterol	*Moringa oleifera*	HaCaT keratinocytes and J774A.1 macrophages	7.5 to 30 μM	24 h	Significant reduction in NLRP3 expression, inhibition of caspase-1, and NF-KB activation in macrophages.	[122]
Polysaccharides	Polysaccharide composed of galactose, glucose, mannose, and arabinose with molar ratios of 5.79:5.77:3.45:1.20 (average MW 63000)	*Trametes orientalis*	Male Kunming mice (induced lung injury)	Intragastrally 50, 100, 200 mg/kg of BW	21 days	Suppression of IL-1β expression and blockage of NLRP3, ASC, and caspase-1 increases in a dose-dependent manner.	[123]
	Polysaccharides extracted from *Dendrobium officinale*	*Dendrobium o* *fficinale*	1. DSS male BalB/c mice (induced induced acute ulcerative colitis) 2. LPS-stimulated NCM460 cells	1. 50, 100, and 200 mg/kg of BW 2. 50, 100, and 200 mg/mL	1. 7 days 2. 24 h	Suppression of NLRP3, ASC, caspase-1, IL-1β, and IL-18 mRNA expression.	[124]
	CYP-1 mannoglucan	*Chinese yam*	1. In vitro RAW 264.7 murine macrophage cells 2. In vivo DDS-induced colitis male C57BL/6 J mice	1. 31.25, 62.5, 125, 250, and 500 μg/mL 2. Intragastrically 300 mg/kg of BW	1. 24 h 2. 7 days	Suppress the expression of several key genes involved in colonic inflammatory signaling pathways (such as NF-κB and NLRP3).	[125]
	*Ganoderma lucidum* polysaccharides	*Ganoderma lucidum*	Male Kun-Ming mice	50, 100, 150 mg/kg of BW	7 days	Decreased protein expression levels of NLRP3, ASC, and caspase-1 in liver tissue.	[126]
	Mannose, arabinose, and fucose at a molar ratio of 1.6:1.0:2.7	*Armillariella tabescens*	Male C57BL/6 J mice	100, 200, and 400 mg/kg Per day	4 weeks	Reduction in MDA, pro-inflammatory factors (TNF-α, IL-18, and IL-1β) and FAS, G6Pase, and PEPCK levels in a dose-dependent manner. Decreased TXNIP and NLRP3 expression levels.	[127]
	Low methoxyl pectin		4-week-old female non-obese diabetic (NOD) mice	Diets with 5% (wt/wt) LMP	1. 36 weeks 2. 18 weeks	Suppression of NLRP3 and associated proteins expression (NLRP3, caspase-1-p20, cleaved IL-1β, and cleaved IL-18) in cecum; increase in the SCFAs (short chain fatty acids) by gut microbiota.	1. [128] 2. [129]
	Non-digestible carbohydrates (NDCs) consisting of pectic homogalacturonan and highly branched rhamnogalacturonan-II, as well as hemicellulosic material including glucomannan, xyloglucan, and glucurono(arabino)xylan	Chayote fruit	Human THP-1 macrophage-like cells Human monocytic cell line THP-1	100, 200, and 400 μg/mL	24 h	Inhibition of CC-induced active caspase-1 (400 μg/mL), reduction in ROS accumulation and IL-1β. mRNA expression of IL-1β and NLRP3 in macrophage-like cells. Inhibition of NLRP3 and IL-1β gene expression in both CC-pretreated macrophage-like cells LPS-induced cells.	[130]
	Mannoglucan sulfate SF-2	Starfish (*A. rollestoni*)	1. RAW 264.7 cells murine macrophages 2. Primary peritoneal macrophages isolated form male ICR mice 3. ICR mice	1. and 2. 80 μg/mL 3. 30 and 60 mg/kg of BW	1. and 2. 0 h, 0.5 h, 1 h, 3 h, 6 h, and 9 h. C. 14 days	Improved release of cytokines and NLRP3 expression by the elevated expression of NLRP3, cleaved caspase-1, and ASC proteins.	[131]

Abbreviations: NLR family pyrin domain containing 3 (NLRP3), caspase-recruitment domain (ASC), interleukin (IL), reactive oxygen species (ROS), tumor necrosis factor (TNF).

## Data Availability

Not applicable.
